# *ADAMTS18* as a candidate gene linking social stress and depression: a cross-species study in African wild dogs (*Lycaon pictus*) and humans

**DOI:** 10.3389/fnbeh.2026.1878769

**Published:** 2026-07-29

**Authors:** Jihoon Park, Jin-Gwan Jeon, Youbin Kang, Kyu-Man Han, Daun Shin, Mi-Ryung Han, Byung-Joo Ham

**Affiliations:** 1 Department of Psychiatry, Korea University Anam Hospital, Korea University College of Medicine, Seoul, Republic of Korea; 2 Division of Life Sciences, College of Life Sciences and Bioengineering, Incheon National University, Incheon, Republic of Korea; 3 Department of Biomedical Sciences, Korea University College of Medicine, Seoul, Republic of Korea; 4 Department of Psychiatry, Asan Medical Center, Seoul, Republic of Korea; 5 Institute for New Drug Development, College of Life Science and Bioengineering, Incheon National University, Incheon, Republic of Korea

**Keywords:** *ADAMTS18*, cross-species genomics, diffusion tensor imaging, *Lycaon pictus*, major depressive disorder, neuroimaging genetics, white matter connectivity

## Abstract

**Background:**

Major depressive disorder (MDD) is a leading cause of disability; however, its neurogenetic mechanisms remain partially understood. Comparative genomic approaches reveal evolutionarily conserved risk factors, particularly when applied to ecologically valid models. *Lycaon pictus* is a social carnivore whose affiliative behavior has been shaped by natural selection. Since impaired social and affiliative function are a feature of depression, genes selected for social function in this species may serve as candidates for the social-affective dimension of MDD. Here, a comparative approach was integrated with human neuroimaging–genetic analysis, focusing on white matter phenotypes.

**Methods:**

Whole-genome sequencing data from *Lycaon pictus* (*n* = 7) and *Canis lupus* (*n* = 36) were analyzed alongside a human cohort of 367 patients with MDD and 161 healthy controls using rare variant association tests. Across analyses, *ADAMTS18* emerged as a convergent gene of interest. Whole-exome sequencing and diffusion tensor imaging (DTI) were conducted on 234 patients with MDD and 135 healthy controls. Eleven common SNPs within *ADAMTS18* were examined for genotype-by-diagnosis and genotype-by-symptom interactions using tract-based DTI metrics.

**Results:**

SNP rs11643211 showed a nominal genotype × diagnosis interaction on axial diffusivity in the bilateral uncinate fasciculus, with elevated values in A allele carriers with MDD. In symptom-based models, rs1948719 was nominally associated with depression severity in the left cingulum–cingulate gyrus. Although these associations did not survive false discovery rate correction, the nominal effects appeared to cluster within tracts implicated in affective integration and self-referential cognition.

**Conclusion:**

The present study is hypothesis-generating and identifies *ADAMTS18* as a gene worth further study in relation to white matter and depression. Cross-species neurogenomics may offer a useful framework for investigating links among social behavior, genetic variation and psychiatric vulnerability. However, given the small sample sizes and the absence of FDR-significant results, these findings should be interpreted as providing an exploratory framework for identifying candidate markers relevant to MDD.

## 1 Introduction

Major depressive disorder (MDD) is one of the most prevalent psychiatric conditions, with a lifetime prevalence of up to 30% (Moffitt et al., 2010). It is characterized by depressive mood, alterations in vegetative symptoms, cognitive impairments, and social withdrawal (Marx et al., 2023). Although clinically recognized as disturbances in mood and activities of daily living, these manifestations are underpinned by complex, dynamic interactions between genetic predisposition and environmental stressors (Krishnan and Nestler, 2008; Cui et al., 2024).

Although large-scale genome-wide association studies (GWAS) have identified several common genetic variants associated with MDD, these findings account for only a small portion of the disorder’s heritability and provide limited insight into the biological mechanisms linking genetic variation to clinical presentation (Howard et al., 2019; Wray et al., 2018). To address this gap, recent studies have increasingly focused on rare genetic variants identified through next-generation sequencing technologies by integrating neuroimaging with genomic data (Ripke et al., 2013; Sanders et al., 2017; Tian et al., 2024; Zhou et al., 2021).

Within this framework, cross-species studies have become valuable for identifying conserved neural mechanisms involved in affect regulation and social behavior (Fox et al., 2021; Bach, 2022; Kim et al., 2020). Canine models have attention due to their sophisticated social cognition, coevolution with humans, and behavioral diversity across breeds (Karlsson and Lindblad-Toh, 2008; Sutter and Ostrander, 2004; Morrill et al., 2023). Several studies have linked canine traits such as affiliative behavior, fear response, and compulsivity to genes implicated in human psychiatric vulnerability (Morrill et al., 2023; Zapata et al., 2016; Sarviaho et al., 2019; Tang et al., 2014). However, most of these models are shaped by domestication or artificial selection, which may introduce biases that obscure naturally evolved socio-affective traits. Therefore, their translational relevance for modeling responses to emotional and social stress may be limited.

The African wild dog (*Lycaon pictus*) is a highly social carnivore endemic to sub-Saharan Africa (Nowak, 2005). Its complex social ecology makes it an informative species for neurogenomic research on social and affective behavior. Compared with domesticated or laboratory-adapted species, *Lycaon* maintains complex social structures in the wild including stable packs centered on an alpha breeding pair and extensive cooperative caregiving (Robbins and McCreery, 2000; McNutt, 1996; Chen, 2019). Attempts at solitary living typically result in high mortality and anxiety symptoms (Courchamp et al., 2000; Zijlmans and Duchateau, 2019), underscoring the species’ strong reliance on affiliative bonds and group cohesion. Social withdrawal and impaired affiliative processing are among the recognized features of MDD. A highly social species such as *Lycaon* may therefore provide insights into genes shaped by selection on affiliative behavior, which could be relevant to the social-affective dimension of depression.

Importantly, recent comparative neuroanatomical studies indicate that the complexity of *Lycaon*’s social behaviors is not attributable to gross brain volume but may instead arise from the organization and connectivity of specific limbic-prefrontal-sensory circuits (Chengetanai et al., 2020). This aligns with evidence that social and emotional regulation in mammals is mediated by evolutionarily conserved systems, including oxytocin-mediated signaling (Smith et al., 2010; Cavanaugh et al., 2015; Olff et al., 2013), amygdala–prefrontal connectivity (Gangopadhyay et al., 2021; Birn et al., 2014; Yizhar and Levy, 2021; Murray and Fellows, 2022), and limbic–hypothalamic–pituitary–adrenal axis dynamics (McEwen and Gianaros, 2011; Ulrich-Lai and Herman, 2009). These insights suggest that the neural basis of *Lycaon*’s affiliative behavior may involve distributed network architecture, white matter tract integrity and neurochemical modulation.

To translate these comparative insights into a neural phenotype in humans, we focused on white matter microstructure quantified using diffusion tensor imaging (DTI), as the primary neural phenotype. Unlike gray matter volume or cortical thickness, white matter provides a systems-level perspective on communication across distant but functionally integrated regions including amygdala, prefrontal cortex, and hippocampus (Sporns, 2013; Eickhoff et al., 2015; Fields, 2008). DTI-derived metrics such as fractional anisotropy (FA) and radial diffusivity (RD) serve as sensitive proxies for axonal integrity and myelination (Heckel et al., 2015).

To our knowledge, this study is among the first to use *Lycaon pictus* to identify genes that may be under selection on social behavior and to test their possible relevance to human white matter phenotypes associated with depression. This integrative framework, combining genomic and neuroimaging data, offers an exploratory approach for identifying candidate markers relevant to MDD.

## 2 Materials and methods

### 2.1 Human cohort

Between March 2010 and February 2021, we enrolled 367 adults with MDD from the outpatient psychiatric clinic at Korea University Anam Hospital (Seoul, Republic of Korea). Board-certified psychiatrists confirmed diagnoses using the Structured Clinical Interview for DSM-IV Axis I Disorders. Eligibility required age ≥ 19 years and self-reported Korean ancestry for three generations. We excluded individuals with comorbid psychiatric disorders, psychotic features, severe suicidality requiring admission, major medical or neurological illness, or MRI contraindications.

Healthy controls (HCs; *n* = 161; age ≥ 19 years) were recruited via community advertisements and screened with identical procedures. Neuroimaging data were available for 234 patients with MDD and 135 HCs; demographic and clinical details for this imaging cohort have been reported (Oh et al., 2024). Depressive symptoms were quantified with the 17-item Hamilton Depression Rating Scale (HDRS-17) (Hamilton, 1960). All participants provided written informed consent. The study protocol was approved by the Institutional Review Board (IRB numbers: 2009AN0105, 2015AN0009, 2016AN0213, 2017AN0185, and 2019AN0174).

### 2.2 Canine cohort

Datasets for *Lycaon pictus* and *Canis lupus* used in this study were obtained from the Sequence Read Archive (SRA) and European Nucleotide Archive. To minimize sequence-related bias arising from inter-cohort differences between human and canine samples, only those with genomic DNA extracted from blood and sequenced using whole-genome sequencing (WGS) with paired-end reads were included. The final dataset comprised seven *Lycaon pictus* and 36 *Canis lupus*, providing a representative cohort for comparative genomics. Detailed project and sample accession numbers are listed in Supplementary Table S1.

### 2.3 Processing of the sequencing data

**Figure 1:**
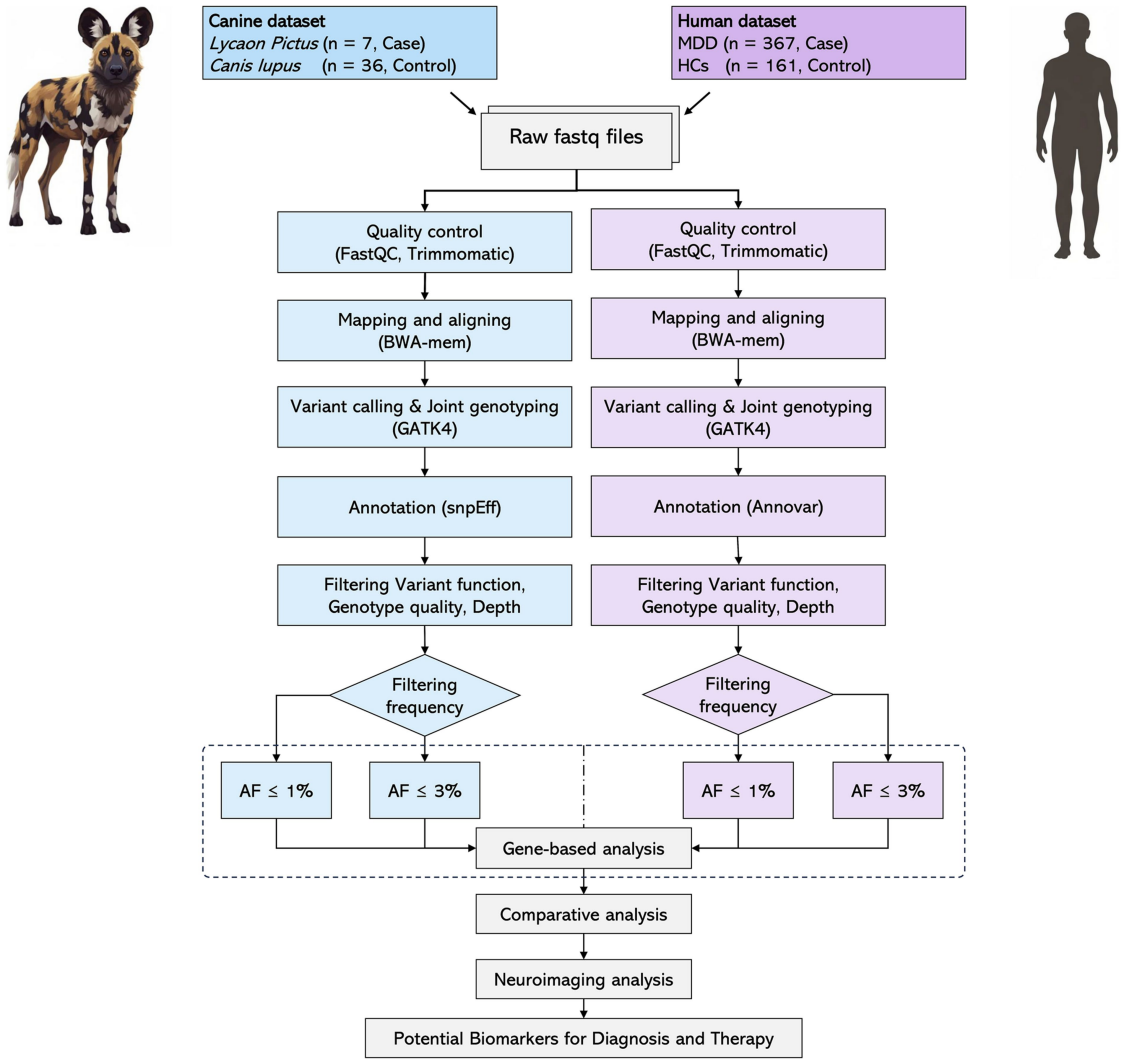
Overview of the analytical workflow used to identify potential biomarkers across two datasets. HCs, healthy controls; MDD, major depressive disorder; AF, allele frequency.

The overall workflow of the cross-species genomic and neuroimaging analyses is summarized in Figure 1. For the human cohort, previously published whole-exome sequencing data of patients with MDD and HCs were used (Oh et al., 2024). Detailed quality control and alignment procedures are described in the same study (Oh et al., 2024). For the canine cohort, WGS data were downloaded from the SRA using SRA Toolkit (v3.1.0, https://github.com/ncbi/sra-tools) with 100–250 bp paired-end reads sequenced using Illumina sequencing technology, including HiSeq 2000, HiSeq 2500, HiSeq 4000, HiSeq X Ten, and NovaSeq 6000. Raw sequencing reads were filtered for low-quality fragments and adapters using Trimmomatic (v0.36) (Bolger et al., 2014), and data quality was assessed using FastQC (v0.12.1, https://github.com/s-andrews/FastQC/releases). The reads were aligned to the CanFam3.1 reference genome using Burrows-Wheeler Aligner MEM (v0.7.17) (Li and Leal, 2008). Duplicate reads were marked, local realignments were performed, and base quality scores were recalibrated using the Genome Analysis Toolkit (GATK, v4.2.0.0) following GATK Best Practices (DePristo et al., 2011). For the downstream analysis, reads with low mapping quality (MAPQ < 20) were removed using SAMtools (v1.10) (Li et al., 2009). Germline short-variant discovery was performed according to GATK Best Practices (v4.2.0.0). HaplotypeCaller in GVCF mode was used to detect insertions, deletions (INDELs), and single-nucleotide polymorphisms (SNPs). CombineGVCFs and GenotypeGVCFs were applied for joint genotyping to identify potential variants at the individual level in the dataset. To filter low-quality SNPs and INDELs, bcftools (v1.10.2) was used to split multi-allelic sites using the CanFam3.1 reference genome. SNPs and INDELs were selected and filtered using SelectVariants and VariantFiltration with the following criteria: QD < 2.0 || FS > 200.0 || ReadPosRankSum < −20.0. Functional consequences of coding-sequence germline variants, including silent and non-silent variants, were predicted using ANNOVAR (v2020) (Wang et al., 2010) and SnpEff (v5.2c) (Cingolani et al., 2012). Human orthologs of the canine-identified genes were determined using the R package biomaRt (v2.58.2) (Durinck et al., 2005; Durinck et al., 2009), which maps functionally matched orthologous genes between canines and humans via the Ensembl database (v104).

### 2.4 Gene-based rare variant association analysis

For both human (MDD vs. HCs) and canine (*Lycaon pictus* vs. *Canis lupus*) cohorts, combined multivariate and collapsing (CMC) (Li and Leal, 2008), variable threshold model by permutation (VT) (Price et al., 2010), sequencing kernel association test (SKAT) (Wu et al., 2011), and optimal sequencing kernel association test (SKATO) (Lee et al., 2012) were performed using Rvtests (v20190205) (Zhan et al., 2016). Principal component analysis (PCA) was applied to condense genetic burden information across genomic regions, using the FastPCA algorithm (Alexander et al., 2009). Rvtests (v20190205) were executed for 1,000,000 permutations with covariates including age, sex, and two principal components. Owing to the absence of age and sex information in the canine cohort, covariates were restricted to the human cohort. Associations of rare coding variants were evaluated using two distinct minor allele frequency (MAF) thresholds: MAF ≤ 3% and MAF ≤ 1%. Finally, *p*-values from the four gene-based rare variant analyses were then combined using the Aggregated Cauchy Association Test (ACAT) (Liu et al., 2019), followed by a Bonferroni correction to control for multiple testing. ACAT calculates the final test statistic by applying weights to the transformed p-values, which are converted using the Cauchy distribution ( $\tan\{(0.5-P_{i})\pi \}$ ).

### 2.5 Neuroimaging acquisition and tract-based preprocessing

DTI was acquired from 234 patients with MDD and 135 HCs using a 3.0 Tesla Trio™ whole-body MRI scanner (Siemens Healthcare GmbH, Erlangen, Germany) at the Korea University MRI Center. Detailed imaging parameters are provided in the Supplementary Methods. Preprocessing was performed using the TRACULA pipeline (FreeSurfer v7.2) to reconstruct 18 bilateral white matter tracts via global probabilistic tractography constrained by anatomical priors (Yendiki et al., 2011; Zollei et al., 2019). Four DTI metrics, FA, mean diffusivity (MD), RD, and axial diffusivity (AD), were extracted for each tract.

### 2.6 Variant selection in *ADAMTS18*

Based on gene-based association results, 61 SNPs within the *ADAMTS18* locus were extracted from the human dataset. All variants were binarized using a dominant genetic model (0 = homozygous major allele; 1 = heterozygous or homozygous minor allele). To ensure statistical power and variant reliability, SNPs were filtered according to the following criteria: MAF ≥ 3% and at least five minor allele carriers across the cohort. These thresholds were chosen to mitigate sparsity-induced bias in interaction models while retaining sensitivity to detect moderate-effect variants. As a result, 11 SNPs passed the filtering criteria and were included in downstream neuroimaging–genetic association analyses.

### 2.7 Imaging-genetic association analyses

To investigate the effects of *ADAMTS18* variants on white matter microstructure, two complementary sets of interaction models were constructed using DTI metrics. First, a tract-wise analysis of covariance (ANCOVA) model was used to test for genotype × diagnosis interaction effects (MDD vs. HC) across the 11 selected SNPs. For each SNP–tract pair, four DTI indices were examined: FA, MD, AD, and RD. All diffusion metrics were z-standardized to enable cross-tract comparison. Second, to assess the moderating effect of depressive symptom severity, linear regression analyses restricted to the MDD group (*n* = 234) were performed. For each SNP–tract pair, the interaction between genotype and HDRS-17 total score on tract-level DTI metrics (FA, MD, AD, and RD) was modeled. Each model included genotype (coded under a dominant model), HDRS score, and their interaction term as predictors.

All interaction models were subjected to false discovery rate (FDR) correction using the Benjamini–Hochberg procedure (*q* < 0.05), with both nominal and FDR-adjusted *p*-values reported. Age and sex were included as covariates in all models. Results are presented in Supplementary Table S2. ANCOVA and linear regression analyses were conducted in Python 3.9 (Van Rossum and Drake, 2009) using the StatsModels library (v0.14.2), and all visualizations were generated in R (R Core Team, 2024; Wickham, 2016).

## 3 Results

### 3.1 Analysis of sequence data for germline variants

Genomes of 367 patients with MDD and 161 HCs were analyzed to detect germline variants, which were subsequently compared with *Lycaon pictus* (*N* = 7) and *Canis lupus* (*N* = 36). In human genomes, an average of 43,911 variants were detected in MDD (26,105 non-silent and 31,145 silent variants), and 43,946 variants were detected in HCs (26,098 non-silent and 31,164 silent variants). In canine genomes, an average of 13,971,558 variants were found in *Lycaon pictus* (79,516 non-silent and 139,031 silent variants) and 4,786,395 variants in *Canis lupus* (24,239 non-silent and 37,276 silent variants). Rare exonic missense variants in humans (MAF < 1%) were selected using data from the 1,000 Genomes Project, Korean Variant Archive (Lee et al., 2017), and Genome Aggregation Database. Canine variants were selected using DogSD (release 2021-05-25, 722 Canine WGS) (Bai et al., 2015; Plassais et al., 2019). Additionally, the orthologs of the canine-identified genes were determined using biomaRt, considering only those mapped one-to-one and excluding genes that were unmapped to any human genes or mapped to multiple human genes. As a result, 15,060 functionally matched orthologous genes were identified for further analysis.

### 3.2 Gene-based rare variants analysis

Four tests (CMC, VT, SKAT, and SKATO) were performed to identify genes associated with depressive behavior using two MAF thresholds (“MAF ≤ 1%” or “MAF ≤ 3%”). The CMC method integrates the advantages of both collapsing and multiple-marker tests, enhancing power and enabling the analysis of rare variants across different MAF categories (Li and Leal, 2008). The VT method applies a variable allele-frequency threshold with permutation testing to detect associations involving both trait-increasing and trait-decreasing variants (Price et al., 2010). The SKAT employs a kernel association test with a variance-component score to analyze rare variants and detect their associations with traits (Wu et al., 2011), making it suitable for testing variants with different effect directions and magnitudes. As an extension of SKAT, the optimal unified test (SKATO) was developed to identify both trait-increasing and trait-decreasing effects by adaptively selecting the optimal linear combination of the burden test and SKAT (Lee et al., 2012). An omnibus testing method, ACAT, was used to combine multiple gene-based methods to improve overall statistical power (Liu et al., 2019). Using these approaches, *ADAMTS18* was identified as a significantly associated gene with MDD in the rare variant group (MAF ≤ 1%). This association was consistently observed in both the human and canine cohorts (*p* = 2.00 × 10^−6^ and *p =* 4.00 × 10^−6^, respectively), as shown in Table 1. However, no significant genes were identified in the uncommon variant group (MAF ≤ 3%).

**Table 1:** Gene-based analysis results for rare variants.

Gene	Human cohort		Canine cohort	
	MAF ≤ 1%	MAF ≤ 3%	MAF ≤ 1%	MAF ≤ 3%
*ADAMTS18*	2.00E-06	6.54E-01	4.00E-06	1.62E-01
Number of genes	4,687	5,370	6,763	7,299
p-value threshold	1.07E-05	9.31E-06	7.39E-06	6.85E-06

Significant *p*-values are indicated in boldface when Bonferroni correction was applied within each group. MAF, minor allele frequency.

### 3.3 Genotype × diagnosis interaction effects on white matter microstructure

**Figure 2:**
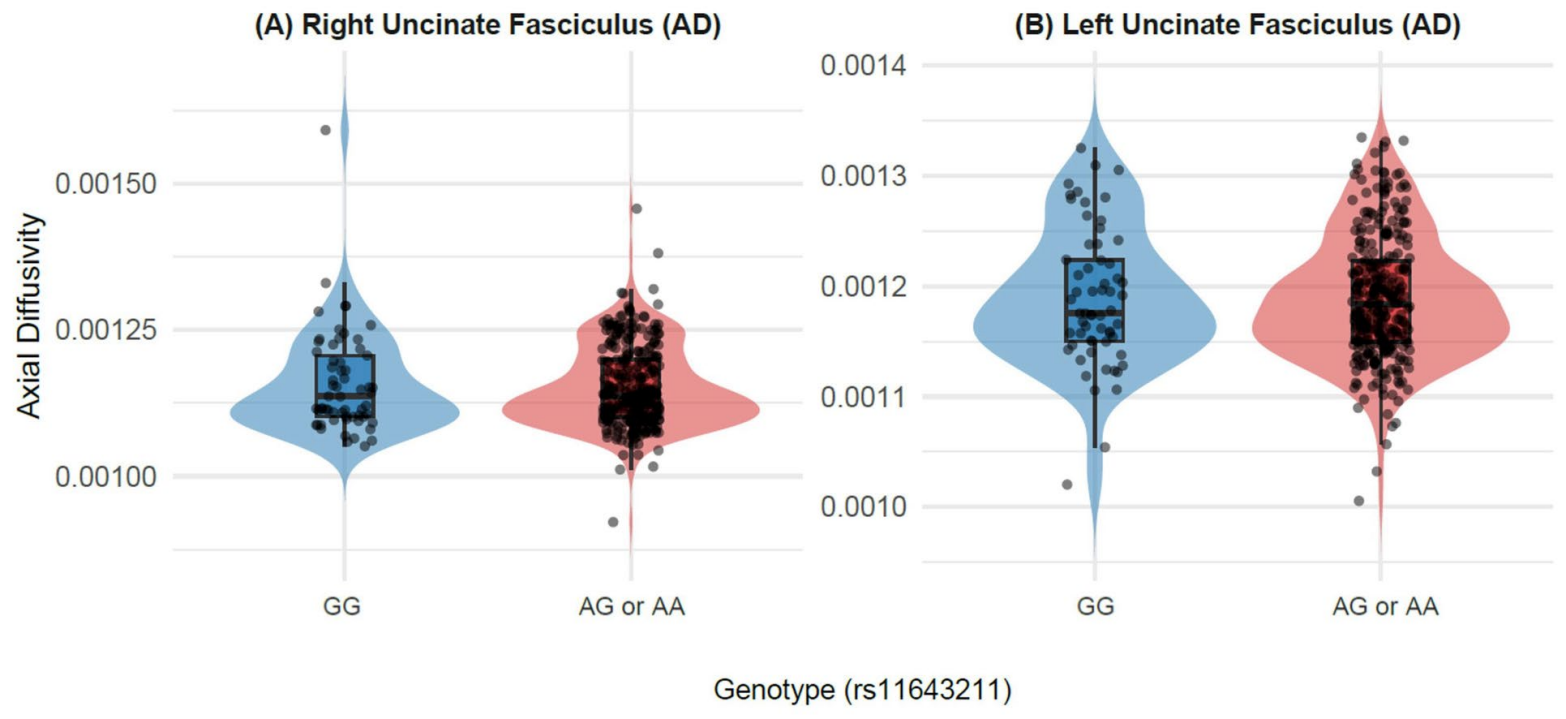
Diagnosis × genotype interaction effects on axial diffusivity in the bilateral uncinate fasciculus. Violin plots illustrate axial diffusivity (AD) values in the left **(A)** and right **(B)** uncinate fasciculus (UNC), stratified by diagnostic group [major depressive disorder (MDD) vs. healthy controls (HC)] and rs11643211 genotype. Each panel displays individual data points overlaid with boxplots and smoothed density curves.

To determine whether *ADAMTS18* variants moderated diagnostic group differences in white matter microstructure, tract-wise ANCOVA analyses were performed to test genotype × diagnosis (MDD vs. HC) interactions on DTI metrics (Table 2). Among the 11 SNPs examined, rs11643211 exhibited the strongest interaction effects on AD in the bilateral uncinate fasciculus (UNC, right: *F* = 7.18, *p* = 7.70 × 10^−3^; left: *F* = 6.42, *p* = 1.17 × 10^−2^). Additional nominally significant interactions were observed for rs11643211 in the left inferior longitudinal fasciculus (ILF, *F* = 4.11, *p =* 4.35 × 10^−2^) and for rs1948719 in the left cingulum–cingulate gyrus (CCG, *F* = 4.44, *p* = 3.57 × 10^−2^). These findings did not survive correction for multiple comparisons. Thus, although the interaction effects were clustered within frontotemporal and limbic association pathways, these results might not provide sufficient evidence for a modulatory role for *ADAMTS18*. Tract-level differences are illustrated in Figure 2 for the UNC, whereas results for the CCG and ILF are presented in Supplementary Figures S1, S2, respectively. Full ANCOVA results across all SNP–tract combinations are provided in Supplementary Table S2.

**Table 2:** ANCOVA results for diagnosis × genotype interaction effects on white matter tract integrity.

Region	SNP	Genomic position (GRCh38)	F	*p*-value
AD RH UNC	rs11643211	Chr16: 77367648	7.18	7.71E-03
AD LH UNC	rs11643211	Chr16: 77367648	6.42	1.17E-02
AD LH CCG	rs1948719	Chr16: 77364184	4.44	3.57E-02
AD LH ILF	rs11643211	Chr16: 77367648	4.11	4.35E-02

Four tract–SNP pairs reached nominal significance (*p* < 0.05) for diagnosis × genotype interaction effects on axial diffusivity (AD). All models included age and sex as covariates, and F statistics with uncorrected p-values are reported. Nominal significance (*p* < 0.05) is shown in bold, but none survived false discovery rate (FDR) correction. Positions are based on human genome build GRCh38. F, degree of freedom; AD, axial diffusivity; LH, left hemisphere; RH, right hemisphere; CCG, cingulum cingulate gyrus; ILF, inferior longitudinal fasciculus; UNC, uncinate fasciculus.

### 3.4 Genotype × depression severity interaction effects on white matter microstructure

To assess whether *ADAMTS18* variants moderated the relationship between depressive symptom severity and white matter structure, interaction effects between HDRS scores and genotype were tested. Linear regression analyses revealed nominally significant HDRS × genotype interactions for rs1948719 in three left hemisphere tracts: the ILF (AD: *β* = 4.70 × 10^−6^, *p* = 3.10 × 10^−3^, *R^2^* = 5.03 × 10^−3^), CCG (AD: *β* = 5.13 × 10^−6^, *p* = 7.00 × 10^−3^, *R^2^* = 5.68 × 10^−2^), and UNC (AD: *β* = 3.08 × 10^−6^, *p* = 2.37 × 10^−2^, *R^2^* = 2.66 × 10^−2^). Notably, although the ILF and UNC showed interaction effects with a different SNP (rs11643211) in the diagnostic group analysis, the left CCG was the only tract demonstrating consistent nominal associations with the same SNP (rs1948719) across both diagnosis- and symptom-level analyses. Additional nominal interactions between depressive symptom severity and genotype were identified in other tracts (Supplementary Table S3), but none survived FDR correction. Although this overlapping signal remained at the uncorrected level, it may indicate a shared locus of vulnerability and support further investigation into the potential role of *ADAMTS18* in white matter structure.

## 4 Discussion

This study integrates rare variant discovery in a non-traditional species with human neuroimaging genetic analysis, examining the relevance of *ADAMTS18* to white matter phenotypes in depression. *Lycaon pictus* is a social carnivore adapted to ecological adversity. We used this species to search for genes that may be shaped by selection on social behavior, subsequently investigating whether any of these genes are relevant to white matter phenotypes in human depression.

SNP rs11643211 in *ADAMTS18* showed a nominal genotype × diagnosis interaction on AD in the bilateral UNCs. Specifically, A-allele carriers with MDD exhibited higher AD values than GG homozygotes and HCs. The UNC, a key tract connecting anterior temporal regions with the orbitofrontal cortex, plays a central role in emotional memory, social evaluation, and affective integration (Phan et al., 2009; Von Der Heide et al., 2013; Xu et al., 2023). Altered AD within this tract may reflect early axonal disorganization or demyelination, potentially serving as a microstructural correlate of emotional dysregulation and rumination (Aung et al., 2013; Alexander et al., 2011; Song et al., 2003). Furthermore, reduced structural integrity of the UNC has been associated with trait vulnerability, maladaptive coping, and affective lability (Xu et al., 2023; Taylor et al., 2007; Hanson et al., 2015). These findings align with those of neuroimaging models that implicate the UNC as a core white matter substrate underlying affective circuit disruption. Although this interaction did not survive FDR correction, its anatomical specificity within the UNC and consistent directionality across diagnostic groups were observed. Imaging genetic associations are highly sensitive to sample size, tract definition, and analytic choices, which can influence the observed effects. Accordingly, our results cannot be interpreted beyond a preliminary signal, and we present the locus as a candidate for further study rather than as an established marker.

Functionally, *ADAMTS18* encodes a secreted metalloproteinase involved in extracellular matrix remodeling and axonal guidance, biological processes that are essential for maintaining white matter architecture (Dubail and Apte, 2015; Apte, 2009). Although this gene has not been widely studied in psychiatric genetics, knockout mouse models have shown reduced depression-like behavior and enhanced dendritic complexity in the prefrontal cortex, suggesting a potential role in stress resilience and synaptic plasticity (Zhu et al., 2019). Moreover, previous GWAS have identified SNPs in *ADAMTS18* (e.g., rs7192208) that are associated with white matter traits, reinforcing its neurodevelopmental relevance (Lopez et al., 2012). Together, these observations are consistent with a possible but unproven role for *ADAMTS18* in white matter organization.

Notably, distinct SNPs within the *ADAMTS18* locus yielded distinct tract-level associations depending on the type of analysis. Although rs11643211 was implicated in diagnosis-based models affecting the UNC, rs1948719 was associated with depressive symptom severity and alterations in the left CCG. The CCG, which integrates self-referential cognition, emotional evaluation, and executive control (Bubb et al., 2018; Van den Heuvel and Sporns, 2013; van den Heuvel et al., 2008; Hu et al., 2024), was the only tract identified across both models. This finding may reflect a shared point of vulnerability, although a multi-pathway effect of *ADAMTS18* requires further investigation.

Additional analyses revealed genotype × symptom interactions in tracts including the superior and inferior longitudinal fasciculus, anterior thalamic radiation, and corticospinal tract. Although these associations did not survive multiple comparison correction, they remain preliminary and may reflect a possible association between *ADAMTS18* variants and depressive symptoms. These findings warrant further investigation in larger cohorts with hypothesis-driven designs.

The broader implications of this study pertain to cross-species psychiatry. In *Lycaon pictus*, individuals experiencing social exclusion or failed dispersal often exhibit behavioral changes such as reduced affiliative behavior and social withdrawal (Courchamp et al., 2000; Edwards et al., 2013; Creel and Creel, 1995). Unlike laboratory animals subjected to artificial stress protocols, *Lycaon* experiences social adversity as a continuous source of natural selection. This ecological setting may provide a biologically relevant context for identifying variants shaped by social interactions, which could be associated with human social-affective function. Whether *ADAMTS18* specifically plays such a role remains to be established. Therefore, rather than positioning *ADAMTS18* as a depression gene, we suggest that it warrants further investigation as a potential contributor to neural responses under environmental adversity.

Our findings are consistent with the emerging perspective that behavioral sophistication in social species is more closely associated with white matter microstructure than with brain volume (Van den Heuvel et al., 2016; Hecht et al., 2013; Rilling et al., 2008). This perspective aligns with human neuroimaging studies which increasingly implicate white matter microstructure, particularly within frontolimbic circuits, as a substrate underlying affective and cognitive vulnerability (Thomason and Thompson, 2011; Bracht et al., 2015; Kieseppä et al., 2010).

Several limitations should be acknowledged. First, gene-based rare variant association testing requires considerably larger sample sizes than those analyzed in the present study. Adequately powered discovery analyses are estimated to require tens of thousands of cases (Zuk et al., 2014; Sazonovs and Barrett, 2018), and large-scale exome studies of affective disorders have not consistently identified significant gene-level associations (Curtis, 2025). Given a human cohort of 367 cases and a canine cohort of seven *Lycaon pictus* genomes, the present analyses were underpowered for definitive rare variant discovery, and the gene-level association for *ADAMTS18* should therefore be regarded as preliminary. Second, none of the neuroimaging genotype interactions survived FDR correction, indicating that these effects are nominal and require replication in larger independent cohorts. Third, as *Lycaon pictus* is an endangered species, the canine data were obtained from publicly available genomes, which precluded behavioral and medical phenotyping and further constrained the available sample size. Fourth, although the comparative framework leverages conserved biological features, intrinsic biological and ecological differences between humans and non-human species limit direct generalizability of cross-species findings. Finally, the analyses relied on a single neuroimaging modality, and imaging genetic associations are themselves sensitive to acquisition, preprocessing and analytic choices, which can introduce bias and limit the robustness of single-modality findings. Collectively, these constraints indicate that the present findings should be interpreted as hypothesis-generating rather than confirmatory.

This study presents an exploratory framework that connects ethologically grounded variant discovery in *Lycaon pictus* with tract-based neuroimaging analyses in humans. Since none of the associations remained significant after multiple-comparison correction, we present this work as a pilot and hypothesis-generating study rather than as evidence that *ADAMTS18* affects white matter or depressive vulnerability. Confirmation in adequately powered samples will be required to establish its relevance to depression. By extending the evolutionary and ecological scope of psychiatric genetics, our framework provides an additional perspective that may inform future work on affective dysregulation. Future studies incorporating multimodal data and larger sample sizes remain essential to validate and expand these findings.

## Data Availability

The datasets presented in this study can be found in online repositories. The names of the repositories and accession numbers can be found in the article/Supplementary material. Human genetic and neuroimaging datasets generated and analyzed during the current study are available from the corresponding author upon reasonable request and subject to institutional ethical and privacy restrictions.
