# Supplementary material for: ADAMTS18 as a candidate gene linking social stress and depression: a cross-species study in African wild dogs (Lycaon pictus) and humans
Source: Front Behav Neurosci. 2026 Jul 29;20:1878769. doi: 10.3389/fnbeh.2026.1878769 (PMC13462048; doi:10.3389/fnbeh.2026.1878769)
Supplement: Supplementary file 1 [file Data_Sheet_1.DOCX]

**Supplementary Materials**

***MRI data acquisition***

Diffusion tensor images were acquired with an echo-planar imaging (EPI) sequence (repetition time = 6300 ms, echo time = 84 ms; field of view = 230 mm; matrix = 128 × 128; slice thickness = 3 mm with no gap; transverse orientation; voxel size = 1.8 × 1.8 × 3.0 mm). Diffusion weighting was applied along 20 directions with one B0 image across 50 slices (b = 0 and 600 s/mm²). Parallel imaging used iPAT–GRAPPA with an acceleration factor of 2, 38 phase-encoding reference lines, and 6/8 partial Fourier in the phase direction. All datasets were visually inspected for scanner- or motion-related artifacts, and participants were rescanned when artifacts were detected.

**Supplementary Table S1. Metadata of whole genome sequences from 7 *Lycaon pictus* and 36 *Canis lupus***

| Sample ID | Scientific name | Bioproject | Biosample | BioExperiment | Platform | Sex |
| --- | --- | --- | --- | --- | --- | --- |
| Ananku | *Lycaon pictus* | PRJNA304992 | SAMN04312208 | SRR2971425 | Illumina HiSeq 2500 | Male |
| WDF20 | *Lycaon pictus* | PRJNA304992 | SAMN04312209 | SRR2971441 | Illumina HiSeq 2500 | Female |
| Sister 2 | *Lycaon pictus* | PRJNA488046 | SAMN09917479 | SRR7874816 | HiSeq X Ten | Female |
| Eureka | *Lycaon pictus* | PRJNA488046 | SAMN09917480 | SRR7874815 | HiSeq X Ten | Female |
| Sister 1 | *Lycaon pictus* | PRJNA488046 | SAMN09917439 | SRR7874817 | Illumina HiSeq 4000 | Female |
| PIC006558 | *Lycaon pictus* | PRJNA648123 | SAMN19522958 | SRR14750369 | Illumina HiSeq 2000 | Female |
| PIC006566 | *Lycaon pictus* | PRJNA648123 | SAMN19522959 | SRR14750370 | Illumina HiSeq 2000 | Male |
| GW1 | *Canis lupus* | PRJNA358192 | SAMN06167395 | SRR5177168 | Illumina HiSeq 2500 | NA |
| GW2 | *Canis lupus* | PRJNA358192 | SAMN06167396 | SRR5177167 | Illumina HiSeq 2500 | NA |
| Gray wolf | *Canis lupus* | PRJNA417738 | SAMN06167396 | SRR6324295 | Illumina HiSeq 2500 | Male |
| Timber wolf | *Canis lupus* | PRJNA417738 | SAMN08005342 | SRR6324294 | Illumina HiSeq 2500 | Female |
| Timber wolf | *Canis lupus* | PRJNA417738 | SAMN08005342 | SRR6324297 | Illumina HiSeq 2500 | Male |
| LUPZCHN00002 | *Canis lupus* | PRJNA448733 | SAMN03168393 | SRR7107845 | Illumina HiSeq 2000 | NA |
| LUPWCHN00003 | *Canis lupus* | PRJNA448733 | SAMN03168394 | SRR7107846 | Illumina HiSeq 2000 | NA |
| LUPZCHN00005 | *Canis lupus* | PRJNA448733 | SAMN03168395 | SRR7107847 | Illumina HiSeq 2000 | NA |
| LUPZCHN00006 | *Canis lupus* | PRJNA448733 | SAMN03168396 | SRR7107848 | Illumina HiSeq 2000 | NA |
| LUPWCHN00008 | *Canis lupus* | PRJNA448733 | SAMN03168397 | SRR7107849 | Illumina HiSeq 2000 | NA |
| LUPWCHN00009 | *Canis lupus* | PRJNA448733 | SAMN03168398 | SRR7107850 | Illumina HiSeq 2000 | NA |
| LUPWCHN00010 | *Canis lupus* | PRJNA448733 | SAMN03168399 | SRR7107851 | Illumina HiSeq 2000 | NA |
| LUPWCHN00013 | *Canis lupus* | PRJNA448733 | SAMN03168400 | SRR7107852 | Illumina HiSeq 2000 | NA |
| RKW13451 | *Canis lupus* | PRJNA448733 | SAMN03366711 | SRR7107871 | Illumina HiSeq 2000 | Female |
| RKW3919 | *Canis lupus* | PRJNA448733 | SAMN03366712 | SRR7107872 | Illumina HiSeq 2000 | Female |
| Sample im5 | *Canis lupus* | PRJNA448733 | SAMN06219538 | SRR7107987 | Illumina HiSeq 2000 | NA |
| Sample ti3 | *Canis lupus* | PRJNA448733 | SAMN06219539 | SRR7107988 | Illumina HiSeq 2000 | NA |
| RSRW021 | *Canis lupus* | PRJNA543877 | SAMN11787846 | SRR9095639 | Illumina HiSeq 2500 | Male |
| RSRW025 | *Canis lupus* | PRJNA543877 | SAMN11787847 | SRR9095638 | Illumina HiSeq 4000 | Male |
| RSRW022 | *Canis lupus* | PRJNA543877 | SAMN11787848 | SRR9095637 | Illumina HiSeq 4000 | Male |
| RSRW023 | *Canis lupus* | PRJNA543877 | SAMN11787849 | SRR9095636 | Illumina HiSeq 4000 | Female |
| RSRW024 | *Canis lupus* | PRJNA543877 | SAMN11787850 | SRR9095635 | Illumina HiSeq 4000 | Female |
| MYL | *Canis lupus* | PRJNA559966 | SAMN12565073 | SRR11085395 | Illumina HiSeq 2500 | NA |
| LXLZ | *Canis lupus* | PRJNA559966 | SAMN12565074 | SRR11085399 | Illumina HiSeq 2500 | NA |
| Mame_Shiba_Inu | *Canis lupus* | PRJNA636107 | SAMN17849183 | SRR13674757 | HiSeq X Ten | NA |
| LUP006630 | *Canis lupus* | PRJNA648123 | SAMN19522949 | SRR14750359 | Illumina HiSeq 2000 | Female |
| LUP006628 | *Canis lupus* | PRJNA648123 | SAMN19522963 | SRR14750375 | Illumina HiSeq 2000 | Female |
| Indian wolf1 | *Canis lupus* | PRJNA714797 | SAMN17169709 | SRR13985171 | Illumina HiSeq 2000 | Male |
| GKSn1 | *Canis lupus* | PRJNA843534 | SAMN28746010 | SRR22387070 | Illumina NovaSeq 6000 | Male |
| GKSp2 | *Canis lupus* | PRJNA843534 | SAMN28746011 | SRR22387069 | Illumina NovaSeq 6000 | Female |
| GKC3 | *Canis lupus* | PRJNA843534 | SAMN28746012 | SRR22387068 | Illumina NovaSeq 6000 | Male |
| WLF001A1 | *Canis lupus* | PRJNA907518 | SAMN31987473 | SRR22520743 | HiSeq X Ten | Male |
| WLF002A1 | *Canis lupus* | PRJNA907518 | SAMN31987474 | SRR22520742 | HiSeq X Ten | Male |
| WLF021A1P | *Canis lupus* | PRJNA907518 | SAMN31987475 | SRR22520741 | HiSeq X Ten | Male |
| WLF022A1P | *Canis lupus* | PRJNA907518 | SAMN31987476 | SRR22520740 | HiSeq X Ten | Female |
| wolf_paternal | *Canis lupus* | PRJNA953635 | SAMN34121418 | SRR24111672 | Illumina NovaSeq 6000 | Male |

Abbreviations: NA, not available

**Supplementary Table S2. ANCOVA results for Diagnosis × Genotype interaction effects on white matter tract integrity**

| Region | SNP | Genomic position  (GRCh38) | F | P-value |
| --- | --- | --- | --- | --- |
| AD RH UNC | rs11643211 | Chr16 : 77367648 | 7.18 | **0.008** |
| AD LH UNC | rs11643211 | Chr16 : 77367649 | 6.42 | **0.012** |
| AD LH CCG | rs1948719 | Chr16 : 77364184 | 4.44 | **0.036** |
| AD LH ILF | rs11643211 | Chr16 : 77367648 | 4.11 | **0.043** |
| AD LH SLFT | rs11643211 | Chr16 : 77367649 | 3.83 | 0.051 |
| FA RH SLFT | rs35296483 | Chr16 : 77293121 | 3.61 | 0.058 |
| FA RH SLFT | rs12935229 | Chr16 : 77294998 | 3.61 | 0.058 |
| FA RH SLFT | rs12935394 | Chr16 : 77295093 | 3.61 | 0.058 |
| AD FMinor | rs11643211 | Chr16 : 77367649 | 3.57 | 0.060 |
| MD RH UNC | rs35296483 | Chr16 : 77293121 | 3.45 | 0.064 |
| MD RH UNC | rs12935229 | Chr16 : 77294998 | 3.45 | 0.064 |
| MD RH UNC | rs12935394 | Chr16 : 77295093 | 3.45 | 0.064 |
| AD RH CAB | rs35296483 | Chr16 : 77293121 | 3.36 | 0.068 |
| AD RH CAB | rs12935229 | Chr16 : 77294998 | 3.36 | 0.068 |
| AD RH CAB | rs12935394 | Chr16 : 77295093 | 3.36 | 0.068 |
| FA FMinor | rs11643211 | Chr16 : 77367649 | 3.34 | 0.069 |
| FA LH SLFT | rs35478105 | Chr16 : 77291428 | 3.31 | 0.070 |
| AD RH UNC | rs35296483 | Chr16 : 77293121 | 3.26 | 0.072 |
| AD RH UNC | rs12935229 | Chr16 : 77294998 | 3.26 | 0.072 |
| AD RH UNC | rs12935394 | Chr16 : 77295093 | 3.26 | 0.072 |
| MD LH ILF | rs1948719 | Chr16 : 77364184 | 3.25 | 0.072 |
| AD RH SLFP | rs11643211 | Chr16 : 77367649 | 3.18 | 0.076 |
| FA LH CAB | rs3743749 | Chr16 : 77289338 | 3.15 | 0.077 |
| MD RH UNC | rs11643211 | Chr16 : 77367649 | 3.13 | 0.078 |
| AD RH ILF | rs11643211 | Chr16 : 77367649 | 3.11 | 0.079 |
| AD FMinor | rs1948719 | Chr16 : 77364184 | 3.03 | 0.083 |
| FA LH ILF | rs35478105 | Chr16 : 77291428 | 2.93 | 0.088 |
| AD LH ILF | rs1948719 | Chr16 : 77364184 | 2.87 | 0.091 |
| AD FMajor | rs1948719 | Chr16 : 77364184 | 2.81 | 0.094 |
| RD RH SLFP | rs3743749 | Chr16 : 77289338 | 2.78 | 0.096 |
| FA LH UNC | rs11643211 | Chr16 : 77367649 | 2.77 | 0.097 |
| AD LH ATR | rs1948719 | Chr16 : 77364184 | 2.76 | 0.098 |
| RD RH UNC | rs35296483 | Chr16 : 77293121 | 2.69 | 0.102 |
| RD RH UNC | rs12935229 | Chr16 : 77294998 | 2.69 | 0.102 |
| RD RH UNC | rs12935394 | Chr16 : 77295093 | 2.69 | 0.102 |
| RD LH ILF | rs1948719 | Chr16 : 77364184 | 2.50 | 0.114 |
| FA LH ATR | rs3743749 | Chr16 : 77289338 | 2.48 | 0.116 |
| FA RH SLFP | rs3743749 | Chr16 : 77289338 | 2.43 | 0.120 |
| AD RH CST | rs11643211 | Chr16 : 77367649 | 2.34 | 0.127 |
| FA LH SLFP | rs35296483 | Chr16 : 77293121 | 2.30 | 0.130 |
| FA LH SLFP | rs12935229 | Chr16 : 77294998 | 2.30 | 0.130 |
| FA LH SLFP | rs12935394 | Chr16 : 77295093 | 2.30 | 0.130 |
| FA LH CST | rs3743749 | Chr16 : 77289338 | 2.25 | 0.134 |
| FA LH CST | rs1948719 | Chr16 : 77364184 | 2.23 | 0.137 |
| FA FMajor | rs1948719 | Chr16 : 77364184 | 2.20 | 0.139 |
| FA RH SLFP | rs35296483 | Chr16 : 77293121 | 2.19 | 0.139 |
| FA RH SLFP | rs12935229 | Chr16 : 77294998 | 2.19 | 0.139 |
| FA RH SLFP | rs12935394 | Chr16 : 77295093 | 2.19 | 0.139 |
| MD LH ILF | rs11643211 | Chr16 : 77367649 | 2.17 | 0.142 |
| AD LH ATR | rs11643211 | Chr16 : 77367649 | 2.16 | 0.142 |
| AD LH CAB | rs35296483 | Chr16 : 77293121 | 2.14 | 0.144 |
| AD LH CAB | rs12935229 | Chr16 : 77294998 | 2.14 | 0.144 |
| AD LH CAB | rs12935394 | Chr16 : 77295093 | 2.14 | 0.144 |
| FA LH ATR | rs35296483 | Chr16 : 77293121 | 2.14 | 0.145 |
| FA LH ATR | rs12935229 | Chr16 : 77294998 | 2.14 | 0.145 |
| FA LH ATR | rs12935394 | Chr16 : 77295093 | 2.14 | 0.145 |
| AD LH SLFT | rs1948719 | Chr16 : 77364184 | 2.06 | 0.152 |
| RD RH SLFT | rs35296483 | Chr16 : 77293121 | 1.98 | 0.160 |
| RD RH SLFT | rs12935229 | Chr16 : 77294998 | 1.98 | 0.160 |
| RD RH SLFT | rs12935394 | Chr16 : 77295093 | 1.98 | 0.160 |
| MD LH ILF | rs35296483 | Chr16 : 77293121 | 1.96 | 0.162 |
| MD LH ILF | rs12935229 | Chr16 : 77294998 | 1.96 | 0.162 |
| MD LH ILF | rs12935394 | Chr16 : 77295093 | 1.96 | 0.162 |
| MD RH ILF | rs11643211 | Chr16 : 77367649 | 1.93 | 0.166 |
| MD RH CAB | rs35296483 | Chr16 : 77293121 | 1.89 | 0.170 |
| MD RH CAB | rs12935229 | Chr16 : 77294998 | 1.89 | 0.170 |
| MD RH CAB | rs12935394 | Chr16 : 77295093 | 1.89 | 0.170 |
| MD LH UNC | rs3743749 | Chr16 : 77289338 | 1.88 | 0.171 |
| AD RH SLFT | rs11643211 | Chr16 : 77367649 | 1.82 | 0.178 |
| AD RH ATR | rs11643211 | Chr16 : 77367649 | 1.79 | 0.182 |
| AD LH CST | rs11643211 | Chr16 : 77367649 | 1.79 | 0.182 |
| RD LH CAB | rs3743749 | Chr16 : 77289338 | 1.78 | 0.183 |
| RD LH UNC | rs3743749 | Chr16 : 77289338 | 1.76 | 0.186 |
| RD LH ATR | rs35296483 | Chr16 : 77293121 | 1.75 | 0.187 |
| RD LH ATR | rs12935229 | Chr16 : 77294998 | 1.75 | 0.187 |
| RD LH ATR | rs12935394 | Chr16 : 77295093 | 1.75 | 0.187 |
| FA RH CST | rs1948719 | Chr16 : 77364184 | 1.75 | 0.187 |
| FA LH UNC | rs35296483 | Chr16 : 77293121 | 1.72 | 0.190 |
| FA LH UNC | rs12935229 | Chr16 : 77294998 | 1.72 | 0.190 |
| FA LH UNC | rs12935394 | Chr16 : 77295093 | 1.72 | 0.190 |
| MD LH UNC | rs1948719 | Chr16 : 77364184 | 1.71 | 0.192 |
| MD RH ATR | rs11643211 | Chr16 : 77367649 | 1.70 | 0.193 |
| AD LH CST | rs1948719 | Chr16 : 77364184 | 1.70 | 0.193 |
| MD RH SLFP | rs3743749 | Chr16 : 77289338 | 1.69 | 0.194 |
| AD LH ILF | rs35296483 | Chr16 : 77293121 | 1.68 | 0.195 |
| AD LH ILF | rs12935229 | Chr16 : 77294998 | 1.68 | 0.195 |
| AD LH ILF | rs12935394 | Chr16 : 77295093 | 1.68 | 0.195 |
| RD LH UNC | rs35296483 | Chr16 : 77293121 | 1.66 | 0.198 |
| RD LH UNC | rs12935229 | Chr16 : 77294998 | 1.66 | 0.198 |
| RD LH UNC | rs12935394 | Chr16 : 77295093 | 1.66 | 0.198 |
| MD LH UNC | rs11643211 | Chr16 : 77367649 | 1.65 | 0.200 |
| MD RH SLFP | rs11643211 | Chr16 : 77367649 | 1.60 | 0.207 |
| AD LH SLFP | rs11643211 | Chr16 : 77367649 | 1.57 | 0.211 |
| RD RH SLFP | rs35296483 | Chr16 : 77293121 | 1.55 | 0.213 |
| RD RH SLFP | rs12935229 | Chr16 : 77294998 | 1.55 | 0.213 |
| RD RH SLFP | rs12935394 | Chr16 : 77295093 | 1.55 | 0.213 |
| MD RH SLFT | rs11643211 | Chr16 : 77367649 | 1.55 | 0.214 |
| RD LH SLFT | rs35478105 | Chr16 : 77291428 | 1.55 | 0.215 |
| MD LH CCG | rs1948719 | Chr16 : 77364184 | 1.54 | 0.215 |
| RD LH ILF | rs35296483 | Chr16 : 77293121 | 1.54 | 0.215 |
| RD LH ILF | rs12935229 | Chr16 : 77294998 | 1.54 | 0.215 |
| RD LH ILF | rs12935394 | Chr16 : 77295093 | 1.54 | 0.215 |
| FA RH CAB | rs3743749 | Chr16 : 77289338 | 1.53 | 0.216 |
| MD FMinor | rs1948719 | Chr16 : 77364184 | 1.52 | 0.219 |
| RD RH ILF | rs35296483 | Chr16 : 77293121 | 1.49 | 0.223 |
| RD RH ILF | rs12935229 | Chr16 : 77294998 | 1.49 | 0.223 |
| RD RH ILF | rs12935394 | Chr16 : 77295093 | 1.49 | 0.223 |
| FA RH SLFP | rs35478105 | Chr16 : 77291428 | 1.46 | 0.227 |
| FA RH CCG | rs35296483 | Chr16 : 77293121 | 1.44 | 0.231 |
| FA RH CCG | rs12935229 | Chr16 : 77294998 | 1.44 | 0.231 |
| FA RH CCG | rs12935394 | Chr16 : 77295093 | 1.44 | 0.231 |
| MD RH ILF | rs35296483 | Chr16 : 77293121 | 1.44 | 0.231 |
| MD RH ILF | rs12935229 | Chr16 : 77294998 | 1.44 | 0.231 |
| MD RH ILF | rs12935394 | Chr16 : 77295093 | 1.44 | 0.231 |
| AD LH UNC | rs1948719 | Chr16 : 77364184 | 1.40 | 0.238 |
| AD RH CCG | rs35478105 | Chr16 : 77291428 | 1.40 | 0.238 |
| RD LH CST | rs3743749 | Chr16 : 77289338 | 1.38 | 0.241 |
| MD LH SLFT | rs11643211 | Chr16 : 77367649 | 1.37 | 0.243 |
| FA LH SLFT | rs35296483 | Chr16 : 77293121 | 1.35 | 0.245 |
| FA LH SLFT | rs12935229 | Chr16 : 77294998 | 1.35 | 0.245 |
| FA LH SLFT | rs12935394 | Chr16 : 77295093 | 1.35 | 0.245 |
| AD LH CAB | rs35478105 | Chr16 : 77291428 | 1.35 | 0.245 |
| RD LH SLFP | rs3743749 | Chr16 : 77289338 | 1.35 | 0.246 |
| FA LH SLFP | rs35478105 | Chr16 : 77291428 | 1.34 | 0.248 |
| MD LH ATR | rs11643211 | Chr16 : 77367649 | 1.31 | 0.254 |
| AD RH CAB | rs11643211 | Chr16 : 77367649 | 1.31 | 0.254 |
| AD FMinor | rs2650907 | Chr16 : 77359310 | 1.26 | 0.262 |
| RD LH UNC | rs1948719 | Chr16 : 77364184 | 1.25 | 0.264 |
| AD RH CST | rs1948719 | Chr16 : 77364184 | 1.24 | 0.266 |
| RD LH SLFT | rs3743749 | Chr16 : 77289338 | 1.20 | 0.273 |
| AD LH UNC | rs3743749 | Chr16 : 77289338 | 1.20 | 0.275 |
| FA RH SLFT | rs35478105 | Chr16 : 77291428 | 1.19 | 0.276 |
| FA RH ILF | rs35296483 | Chr16 : 77293121 | 1.15 | 0.283 |
| FA RH ILF | rs12935229 | Chr16 : 77294998 | 1.15 | 0.283 |
| FA RH ILF | rs12935394 | Chr16 : 77295093 | 1.15 | 0.283 |
| RD RH ATR | rs11643211 | Chr16 : 77367649 | 1.15 | 0.284 |
| RD RH CCG | rs35296483 | Chr16 : 77293121 | 1.15 | 0.284 |
| RD RH CCG | rs12935229 | Chr16 : 77294998 | 1.15 | 0.284 |
| RD RH CCG | rs12935394 | Chr16 : 77295093 | 1.15 | 0.284 |
| MD LH ATR | rs1948719 | Chr16 : 77364184 | 1.10 | 0.296 |
| MD LH UNC | rs35296483 | Chr16 : 77293121 | 1.09 | 0.296 |
| MD LH UNC | rs12935229 | Chr16 : 77294998 | 1.09 | 0.296 |
| MD LH UNC | rs12935394 | Chr16 : 77295093 | 1.09 | 0.296 |
| RD RH SLFT | rs11643211 | Chr16 : 77367649 | 1.07 | 0.302 |
| MD RH SLFP | rs35296483 | Chr16 : 77293121 | 1.05 | 0.307 |
| MD RH SLFP | rs12935229 | Chr16 : 77294998 | 1.05 | 0.307 |
| MD RH SLFP | rs12935394 | Chr16 : 77295093 | 1.05 | 0.307 |
| MD LH CAB | rs35478105 | Chr16 : 77291428 | 1.04 | 0.309 |
| MD RH CST | rs11643211 | Chr16 : 77367649 | 1.03 | 0.310 |
| MD RH UNC | rs1948719 | Chr16 : 77364184 | 1.03 | 0.312 |
| RD RH CAB | rs35296483 | Chr16 : 77293121 | 1.03 | 0.312 |
| RD RH CAB | rs12935229 | Chr16 : 77294998 | 1.03 | 0.312 |
| RD RH CAB | rs12935394 | Chr16 : 77295093 | 1.03 | 0.312 |
| AD RH ILF | rs1948719 | Chr16 : 77364184 | 1.02 | 0.314 |
| MD LH SLFP | rs3743749 | Chr16 : 77289338 | 1.02 | 0.314 |
| MD RH SLFT | rs35296483 | Chr16 : 77293121 | 1.00 | 0.318 |
| MD RH SLFT | rs12935229 | Chr16 : 77294998 | 1.00 | 0.318 |
| MD RH SLFT | rs12935394 | Chr16 : 77295093 | 1.00 | 0.318 |
| AD FMajor | rs3743749 | Chr16 : 77289338 | 0.99 | 0.320 |
| MD LH CAB | rs3743749 | Chr16 : 77289338 | 0.98 | 0.322 |
| RD FMajor | rs35478105 | Chr16 : 77291428 | 0.97 | 0.325 |
| MD LH SLFT | rs3743749 | Chr16 : 77289338 | 0.96 | 0.327 |
| MD FMajor | rs35296483 | Chr16 : 77293121 | 0.93 | 0.335 |
| MD FMajor | rs12935229 | Chr16 : 77294998 | 0.93 | 0.335 |
| MD FMajor | rs12935394 | Chr16 : 77295093 | 0.93 | 0.335 |
| RD LH SLFT | rs35296483 | Chr16 : 77293121 | 0.92 | 0.338 |
| RD LH SLFT | rs12935229 | Chr16 : 77294998 | 0.92 | 0.338 |
| RD LH SLFT | rs12935394 | Chr16 : 77295093 | 0.92 | 0.338 |
| FA RH CAB | rs11643211 | Chr16 : 77367649 | 0.91 | 0.340 |
| RD RH UNC | rs1948719 | Chr16 : 77364184 | 0.90 | 0.343 |
| RD LH ILF | rs35478105 | Chr16 : 77291428 | 0.90 | 0.343 |
| MD LH ATR | rs35296483 | Chr16 : 77293121 | 0.87 | 0.351 |
| MD LH ATR | rs12935229 | Chr16 : 77294998 | 0.87 | 0.351 |
| MD LH ATR | rs12935394 | Chr16 : 77295093 | 0.87 | 0.351 |
| RD RH CAB | rs3743749 | Chr16 : 77289338 | 0.87 | 0.352 |
| AD LH SLFP | rs1948719 | Chr16 : 77364184 | 0.85 | 0.356 |
| RD RH ATR | rs35296483 | Chr16 : 77293121 | 0.84 | 0.361 |
| RD RH ATR | rs12935229 | Chr16 : 77294998 | 0.84 | 0.361 |
| RD RH ATR | rs12935394 | Chr16 : 77295093 | 0.84 | 0.361 |
| RD RH UNC | rs11643211 | Chr16 : 77367649 | 0.84 | 0.361 |
| FA LH SLFP | rs3743749 | Chr16 : 77289338 | 0.82 | 0.366 |
| AD RH UNC | rs1948719 | Chr16 : 77364184 | 0.82 | 0.366 |
| FA RH CAB | rs2650907 | Chr16 : 77359310 | 0.81 | 0.370 |
| RD LH ATR | rs3743749 | Chr16 : 77289338 | 0.81 | 0.370 |
| MD LH CCG | rs11643211 | Chr16 : 77367649 | 0.80 | 0.372 |
| RD FMajor | rs35296483 | Chr16 : 77293121 | 0.80 | 0.372 |
| RD FMajor | rs12935229 | Chr16 : 77294998 | 0.80 | 0.372 |
| RD FMajor | rs12935394 | Chr16 : 77295093 | 0.80 | 0.372 |
| RD RH ILF | rs35478105 | Chr16 : 77291428 | 0.80 | 0.372 |
| AD RH ILF | rs35296483 | Chr16 : 77293121 | 0.80 | 0.372 |
| AD RH ILF | rs12935229 | Chr16 : 77294998 | 0.80 | 0.372 |
| AD RH ILF | rs12935394 | Chr16 : 77295093 | 0.80 | 0.372 |
| MD LH SLFP | rs11643211 | Chr16 : 77367649 | 0.80 | 0.372 |
| FA LH UNC | rs3743749 | Chr16 : 77289338 | 0.78 | 0.377 |
| MD RH ILF | rs1948719 | Chr16 : 77364184 | 0.78 | 0.378 |
| FA LH ATR | rs1948719 | Chr16 : 77364184 | 0.78 | 0.379 |
| RD LH SLFP | rs35296483 | Chr16 : 77293121 | 0.77 | 0.380 |
| RD LH SLFP | rs12935229 | Chr16 : 77294998 | 0.77 | 0.380 |
| RD LH SLFP | rs12935394 | Chr16 : 77295093 | 0.77 | 0.380 |
| AD RH SLFT | rs1948719 | Chr16 : 77364184 | 0.77 | 0.380 |
| MD LH SLFT | rs1948719 | Chr16 : 77364184 | 0.77 | 0.380 |
| AD RH ATR | rs1948719 | Chr16 : 77364184 | 0.77 | 0.382 |
| FA RH CST | rs35478105 | Chr16 : 77291428 | 0.76 | 0.383 |
| MD FMinor | rs35296483 | Chr16 : 77293121 | 0.76 | 0.384 |
| MD FMinor | rs12935229 | Chr16 : 77294998 | 0.76 | 0.384 |
| MD FMinor | rs12935394 | Chr16 : 77295093 | 0.76 | 0.384 |
| RD RH ILF | rs11643211 | Chr16 : 77367649 | 0.76 | 0.385 |
| AD LH CAB | rs2650907 | Chr16 : 77359310 | 0.75 | 0.387 |
| RD LH CAB | rs35478105 | Chr16 : 77291428 | 0.73 | 0.394 |
| RD RH CST | rs35296483 | Chr16 : 77293121 | 0.72 | 0.396 |
| RD RH CST | rs12935229 | Chr16 : 77294998 | 0.72 | 0.396 |
| RD RH CST | rs12935394 | Chr16 : 77295093 | 0.72 | 0.396 |
| FA RH SLFT | rs1948719 | Chr16 : 77364184 | 0.72 | 0.396 |
| MD RH CST | rs35296483 | Chr16 : 77293121 | 0.71 | 0.399 |
| MD RH CST | rs12935229 | Chr16 : 77294998 | 0.71 | 0.399 |
| MD RH CST | rs12935394 | Chr16 : 77295093 | 0.71 | 0.399 |
| FA FMajor | rs35478105 | Chr16 : 77291428 | 0.71 | 0.400 |
| FA LH CCG | rs1948719 | Chr16 : 77364184 | 0.71 | 0.400 |
| RD RH SLFT | rs3743749 | Chr16 : 77289338 | 0.70 | 0.404 |
| FA RH CAB | rs1948719 | Chr16 : 77364184 | 0.69 | 0.408 |
| FA RH ATR | rs35296483 | Chr16 : 77293121 | 0.68 | 0.409 |
| FA RH ATR | rs12935229 | Chr16 : 77294998 | 0.68 | 0.409 |
| FA RH ATR | rs12935394 | Chr16 : 77295093 | 0.68 | 0.409 |
| FA RH ILF | rs35478105 | Chr16 : 77291428 | 0.68 | 0.409 |
| RD RH SLFP | rs11643211 | Chr16 : 77367649 | 0.68 | 0.409 |
| RD LH ILF | rs11643211 | Chr16 : 77367649 | 0.68 | 0.409 |
| MD LH CST | rs11643211 | Chr16 : 77367649 | 0.68 | 0.411 |
| MD LH CAB | rs35296483 | Chr16 : 77293121 | 0.67 | 0.413 |
| MD LH CAB | rs12935229 | Chr16 : 77294998 | 0.67 | 0.413 |
| MD LH CAB | rs12935394 | Chr16 : 77295093 | 0.67 | 0.413 |
| MD LH SLFT | rs35478105 | Chr16 : 77291428 | 0.67 | 0.414 |
| AD FMinor | rs35296483 | Chr16 : 77293121 | 0.67 | 0.415 |
| AD FMinor | rs12935229 | Chr16 : 77294998 | 0.67 | 0.415 |
| AD FMinor | rs12935394 | Chr16 : 77295093 | 0.67 | 0.415 |
| MD LH CAB | rs2650907 | Chr16 : 77359310 | 0.65 | 0.420 |
| FA RH CST | rs35296483 | Chr16 : 77293121 | 0.65 | 0.421 |
| FA RH CST | rs12935229 | Chr16 : 77294998 | 0.65 | 0.421 |
| FA RH CST | rs12935394 | Chr16 : 77295093 | 0.65 | 0.421 |
| FA LH CAB | rs35296483 | Chr16 : 77293121 | 0.64 | 0.423 |
| FA LH CAB | rs12935229 | Chr16 : 77294998 | 0.64 | 0.423 |
| FA LH CAB | rs12935394 | Chr16 : 77295093 | 0.64 | 0.423 |
| MD LH SLFT | rs35296483 | Chr16 : 77293121 | 0.64 | 0.424 |
| MD LH SLFT | rs12935229 | Chr16 : 77294998 | 0.64 | 0.424 |
| MD LH SLFT | rs12935394 | Chr16 : 77295093 | 0.64 | 0.424 |
| FA RH ATR | rs35478105 | Chr16 : 77291428 | 0.64 | 0.425 |
| RD LH SLFP | rs35478105 | Chr16 : 77291428 | 0.63 | 0.427 |
| MD FMajor | rs35478105 | Chr16 : 77291428 | 0.62 | 0.431 |
| FA RH UNC | rs35296483 | Chr16 : 77293121 | 0.62 | 0.431 |
| FA RH UNC | rs12935229 | Chr16 : 77294998 | 0.62 | 0.431 |
| FA RH UNC | rs12935394 | Chr16 : 77295093 | 0.62 | 0.431 |
| RD RH CCG | rs11643211 | Chr16 : 77367649 | 0.62 | 0.432 |
| AD FMajor | rs35296483 | Chr16 : 77293121 | 0.61 | 0.433 |
| AD FMajor | rs12935229 | Chr16 : 77294998 | 0.61 | 0.433 |
| AD FMajor | rs12935394 | Chr16 : 77295093 | 0.61 | 0.433 |
| FA LH SLFT | rs1948719 | Chr16 : 77364184 | 0.61 | 0.434 |
| MD FMinor | rs2650907 | Chr16 : 77359310 | 0.61 | 0.435 |
| FA RH UNC | rs11643211 | Chr16 : 77367649 | 0.61 | 0.435 |
| RD RH ATR | rs35478105 | Chr16 : 77291428 | 0.60 | 0.437 |
| RD LH CCG | rs11643211 | Chr16 : 77367649 | 0.60 | 0.437 |
| AD FMajor | rs11643211 | Chr16 : 77367649 | 0.60 | 0.440 |
| AD RH UNC | rs2650907 | Chr16 : 77359310 | 0.60 | 0.441 |
| FA LH ATR | rs2650907 | Chr16 : 77359310 | 0.59 | 0.441 |
| MD RH CCG | rs11643211 | Chr16 : 77367649 | 0.59 | 0.441 |
| FA LH SLFT | rs11643211 | Chr16 : 77367649 | 0.57 | 0.450 |
| FA FMinor | rs1948719 | Chr16 : 77364184 | 0.57 | 0.450 |
| MD LH CST | rs35296483 | Chr16 : 77293121 | 0.57 | 0.450 |
| MD LH CST | rs12935229 | Chr16 : 77294998 | 0.57 | 0.450 |
| MD LH CST | rs12935394 | Chr16 : 77295093 | 0.57 | 0.450 |
| RD LH ATR | rs11643211 | Chr16 : 77367649 | 0.57 | 0.451 |
| AD RH CCG | rs35296483 | Chr16 : 77293121 | 0.57 | 0.451 |
| AD RH CCG | rs12935229 | Chr16 : 77294998 | 0.57 | 0.451 |
| AD RH CCG | rs12935394 | Chr16 : 77295093 | 0.57 | 0.451 |
| RD RH SLFT | rs35478105 | Chr16 : 77291428 | 0.57 | 0.452 |
| RD FMinor | rs3743749 | Chr16 : 77289338 | 0.56 | 0.454 |
| RD LH CST | rs35296483 | Chr16 : 77293121 | 0.56 | 0.455 |
| RD LH CST | rs12935229 | Chr16 : 77294998 | 0.56 | 0.455 |
| RD LH CST | rs12935394 | Chr16 : 77295093 | 0.56 | 0.455 |
| FA FMajor | rs3743749 | Chr16 : 77289338 | 0.54 | 0.462 |
| RD RH UNC | rs3743749 | Chr16 : 77289338 | 0.53 | 0.467 |
| RD LH CAB | rs2650907 | Chr16 : 77359310 | 0.51 | 0.477 |
| AD RH CST | rs35296483 | Chr16 : 77293121 | 0.51 | 0.477 |
| AD RH CST | rs12935229 | Chr16 : 77294998 | 0.51 | 0.477 |
| AD RH CST | rs12935394 | Chr16 : 77295093 | 0.51 | 0.477 |
| MD RH ATR | rs35296483 | Chr16 : 77293121 | 0.48 | 0.490 |
| MD RH ATR | rs12935229 | Chr16 : 77294998 | 0.48 | 0.490 |
| MD RH ATR | rs12935394 | Chr16 : 77295093 | 0.48 | 0.490 |
| MD RH ILF | rs35478105 | Chr16 : 77291428 | 0.48 | 0.491 |
| MD LH SLFP | rs1948719 | Chr16 : 77364184 | 0.47 | 0.493 |
| FA FMinor | rs3743749 | Chr16 : 77289338 | 0.47 | 0.494 |
| RD RH ATR | rs3743749 | Chr16 : 77289338 | 0.47 | 0.494 |
| MD RH CAB | rs3743749 | Chr16 : 77289338 | 0.46 | 0.496 |
| MD RH CCG | rs35296483 | Chr16 : 77293121 | 0.46 | 0.498 |
| MD RH CCG | rs12935229 | Chr16 : 77294998 | 0.46 | 0.498 |
| MD RH CCG | rs12935394 | Chr16 : 77295093 | 0.46 | 0.498 |
| FA LH SLFT | rs3743749 | Chr16 : 77289338 | 0.46 | 0.499 |
| AD RH UNC | rs35478105 | Chr16 : 77291428 | 0.45 | 0.502 |
| MD RH CCG | rs35478105 | Chr16 : 77291428 | 0.45 | 0.504 |
| AD LH ILF | rs3743749 | Chr16 : 77289338 | 0.44 | 0.506 |
| MD RH SLFT | rs3743749 | Chr16 : 77289338 | 0.44 | 0.508 |
| FA LH ATR | rs35478105 | Chr16 : 77291428 | 0.44 | 0.510 |
| AD RH CST | rs3743749 | Chr16 : 77289338 | 0.43 | 0.513 |
| AD LH ATR | rs2650907 | Chr16 : 77359310 | 0.43 | 0.514 |
| FA RH CCG | rs11643211 | Chr16 : 77367649 | 0.43 | 0.514 |
| MD LH CST | rs3743749 | Chr16 : 77289338 | 0.43 | 0.514 |
| RD FMinor | rs35296483 | Chr16 : 77293121 | 0.41 | 0.520 |
| RD FMinor | rs12935229 | Chr16 : 77294998 | 0.41 | 0.520 |
| RD FMinor | rs12935394 | Chr16 : 77295093 | 0.41 | 0.520 |
| FA RH ATR | rs3743749 | Chr16 : 77289338 | 0.41 | 0.522 |
| AD LH SLFT | rs3743749 | Chr16 : 77289338 | 0.41 | 0.523 |
| FA LH ILF | rs35296483 | Chr16 : 77293121 | 0.41 | 0.523 |
| FA LH ILF | rs12935229 | Chr16 : 77294998 | 0.41 | 0.523 |
| FA LH ILF | rs12935394 | Chr16 : 77295093 | 0.41 | 0.523 |
| RD RH ILF | rs1948719 | Chr16 : 77364184 | 0.41 | 0.524 |
| AD FMinor | rs35478105 | Chr16 : 77291428 | 0.40 | 0.527 |
| AD RH CAB | rs1948719 | Chr16 : 77364184 | 0.40 | 0.528 |
| MD LH SLFP | rs35478105 | Chr16 : 77291428 | 0.40 | 0.530 |
| AD LH CCG | rs11643211 | Chr16 : 77367649 | 0.40 | 0.530 |
| FA RH ATR | rs1948719 | Chr16 : 77364184 | 0.39 | 0.530 |
| MD RH CST | rs3743749 | Chr16 : 77289338 | 0.39 | 0.530 |
| AD LH SLFP | rs3743749 | Chr16 : 77289338 | 0.39 | 0.532 |
| AD LH CCG | rs2650907 | Chr16 : 77359310 | 0.39 | 0.534 |
| MD FMinor | rs11643211 | Chr16 : 77367649 | 0.38 | 0.536 |
| RD RH SLFP | rs35478105 | Chr16 : 77291428 | 0.38 | 0.536 |
| MD RH UNC | rs3743749 | Chr16 : 77289338 | 0.38 | 0.539 |
| FA LH CST | rs35296483 | Chr16 : 77293121 | 0.38 | 0.540 |
| FA LH CST | rs12935229 | Chr16 : 77294998 | 0.38 | 0.540 |
| FA LH CST | rs12935394 | Chr16 : 77295093 | 0.38 | 0.540 |
| FA FMajor | rs35296483 | Chr16 : 77293121 | 0.38 | 0.541 |
| FA FMajor | rs12935229 | Chr16 : 77294998 | 0.38 | 0.541 |
| FA FMajor | rs12935394 | Chr16 : 77295093 | 0.38 | 0.541 |
| AD LH CST | rs2650907 | Chr16 : 77359310 | 0.37 | 0.541 |
| FA LH CCG | rs11643211 | Chr16 : 77367649 | 0.37 | 0.543 |
| MD LH ILF | rs3743749 | Chr16 : 77289338 | 0.37 | 0.546 |
| RD RH CAB | rs2650907 | Chr16 : 77359310 | 0.36 | 0.547 |
| MD FMinor | rs3743749 | Chr16 : 77289338 | 0.36 | 0.547 |
| AD RH CCG | rs1948719 | Chr16 : 77364184 | 0.36 | 0.548 |
| AD LH CST | rs35296483 | Chr16 : 77293121 | 0.36 | 0.550 |
| AD LH CST | rs12935229 | Chr16 : 77294998 | 0.36 | 0.550 |
| AD LH CST | rs12935394 | Chr16 : 77295093 | 0.36 | 0.550 |
| RD RH CST | rs11643211 | Chr16 : 77367649 | 0.36 | 0.551 |
| FA RH SLFT | rs11643211 | Chr16 : 77367649 | 0.35 | 0.553 |
| MD RH ATR | rs1948719 | Chr16 : 77364184 | 0.35 | 0.553 |
| FA LH ILF | rs1948719 | Chr16 : 77364184 | 0.35 | 0.553 |
| FA LH SLFP | rs2650907 | Chr16 : 77359310 | 0.35 | 0.554 |
| MD RH CCG | rs3743749 | Chr16 : 77289338 | 0.35 | 0.557 |
| MD LH SLFP | rs35296483 | Chr16 : 77293121 | 0.34 | 0.559 |
| MD LH SLFP | rs12935229 | Chr16 : 77294998 | 0.34 | 0.559 |
| MD LH SLFP | rs12935394 | Chr16 : 77295093 | 0.34 | 0.559 |
| RD FMinor | rs11643211 | Chr16 : 77367649 | 0.34 | 0.560 |
| FA RH CAB | rs35296483 | Chr16 : 77293121 | 0.33 | 0.563 |
| FA RH CAB | rs12935229 | Chr16 : 77294998 | 0.33 | 0.563 |
| FA RH CAB | rs12935394 | Chr16 : 77295093 | 0.33 | 0.563 |
| FA RH UNC | rs3743749 | Chr16 : 77289338 | 0.32 | 0.570 |
| FA RH SLFT | rs2650907 | Chr16 : 77359310 | 0.32 | 0.572 |
| AD RH SLFP | rs1948719 | Chr16 : 77364184 | 0.32 | 0.573 |
| AD RH SLFP | rs3743749 | Chr16 : 77289338 | 0.32 | 0.574 |
| MD RH ATR | rs3743749 | Chr16 : 77289338 | 0.32 | 0.575 |
| AD LH UNC | rs35478105 | Chr16 : 77291428 | 0.31 | 0.575 |
| FA LH ILF | rs11643211 | Chr16 : 77367649 | 0.31 | 0.577 |
| RD FMajor | rs1948719 | Chr16 : 77364184 | 0.31 | 0.580 |
| RD RH CST | rs3743749 | Chr16 : 77289338 | 0.30 | 0.582 |
| AD RH SLFP | rs35296483 | Chr16 : 77293121 | 0.30 | 0.585 |
| AD RH SLFP | rs12935229 | Chr16 : 77294998 | 0.30 | 0.585 |
| AD RH SLFP | rs12935394 | Chr16 : 77295093 | 0.30 | 0.585 |
| RD LH SLFP | rs11643211 | Chr16 : 77367649 | 0.30 | 0.586 |
| MD RH CAB | rs2650907 | Chr16 : 77359310 | 0.29 | 0.589 |
| MD RH ATR | rs35478105 | Chr16 : 77291428 | 0.28 | 0.598 |
| FA RH UNC | rs1948719 | Chr16 : 77364184 | 0.28 | 0.600 |
| RD RH CCG | rs3743749 | Chr16 : 77289338 | 0.27 | 0.606 |
| FA RH SLFT | rs3743749 | Chr16 : 77289338 | 0.26 | 0.608 |
| MD RH CAB | rs11643211 | Chr16 : 77367649 | 0.26 | 0.612 |
| RD LH SLFT | rs11643211 | Chr16 : 77367649 | 0.26 | 0.614 |
| MD FMajor | rs11643211 | Chr16 : 77367649 | 0.25 | 0.615 |
| AD LH ILF | rs35478105 | Chr16 : 77291428 | 0.25 | 0.620 |
| FA RH SLFP | rs2650907 | Chr16 : 77359310 | 0.25 | 0.621 |
| RD LH CST | rs1948719 | Chr16 : 77364184 | 0.23 | 0.634 |
| RD LH ATR | rs1948719 | Chr16 : 77364184 | 0.23 | 0.634 |
| AD LH UNC | rs35296483 | Chr16 : 77293121 | 0.22 | 0.636 |
| AD LH UNC | rs12935229 | Chr16 : 77294998 | 0.22 | 0.636 |
| AD LH UNC | rs12935394 | Chr16 : 77295093 | 0.22 | 0.636 |
| AD LH CAB | rs11643211 | Chr16 : 77367649 | 0.22 | 0.640 |
| RD RH ILF | rs2650907 | Chr16 : 77359310 | 0.22 | 0.640 |
| MD RH UNC | rs35478105 | Chr16 : 77291428 | 0.22 | 0.642 |
| RD RH SLFT | rs2650907 | Chr16 : 77359310 | 0.21 | 0.644 |
| RD LH ILF | rs3743749 | Chr16 : 77289338 | 0.21 | 0.647 |
| AD LH UNC | rs2650907 | Chr16 : 77359310 | 0.21 | 0.649 |
| MD RH CCG | rs1948719 | Chr16 : 77364184 | 0.21 | 0.650 |
| AD RH SLFP | rs35478105 | Chr16 : 77291428 | 0.20 | 0.651 |
| FA RH ILF | rs2650907 | Chr16 : 77359310 | 0.20 | 0.654 |
| RD LH SLFP | rs1948719 | Chr16 : 77364184 | 0.20 | 0.656 |
| RD LH SLFP | rs2650907 | Chr16 : 77359310 | 0.19 | 0.659 |
| MD LH ATR | rs3743749 | Chr16 : 77289338 | 0.19 | 0.660 |
| FA LH UNC | rs2650907 | Chr16 : 77359310 | 0.19 | 0.663 |
| RD FMinor | rs1948719 | Chr16 : 77364184 | 0.19 | 0.664 |
| AD RH ILF | rs3743749 | Chr16 : 77289338 | 0.19 | 0.664 |
| FA RH SLFP | rs1948719 | Chr16 : 77364184 | 0.19 | 0.666 |
| MD RH UNC | rs2650907 | Chr16 : 77359310 | 0.18 | 0.668 |
| AD LH SLFT | rs35296483 | Chr16 : 77293121 | 0.18 | 0.669 |
| AD LH SLFT | rs12935229 | Chr16 : 77294998 | 0.18 | 0.669 |
| AD LH SLFT | rs12935394 | Chr16 : 77295093 | 0.18 | 0.669 |
| MD LH SLFP | rs2650907 | Chr16 : 77359310 | 0.18 | 0.670 |
| MD RH SLFT | rs1948719 | Chr16 : 77364184 | 0.18 | 0.672 |
| MD RH SLFT | rs35478105 | Chr16 : 77291428 | 0.18 | 0.673 |
| FA LH UNC | rs35478105 | Chr16 : 77291428 | 0.18 | 0.674 |
| RD RH CST | rs35478105 | Chr16 : 77291428 | 0.17 | 0.677 |
| MD RH SLFT | rs2650907 | Chr16 : 77359310 | 0.17 | 0.682 |
| AD FMajor | rs2650907 | Chr16 : 77359310 | 0.17 | 0.683 |
| MD RH ILF | rs2650907 | Chr16 : 77359310 | 0.17 | 0.683 |
| RD LH CST | rs11643211 | Chr16 : 77367649 | 0.16 | 0.687 |
| RD LH SLFT | rs1948719 | Chr16 : 77364184 | 0.16 | 0.688 |
| FA RH ILF | rs11643211 | Chr16 : 77367649 | 0.16 | 0.689 |
| RD LH CAB | rs1948719 | Chr16 : 77364184 | 0.16 | 0.690 |
| FA RH CST | rs2650907 | Chr16 : 77359310 | 0.16 | 0.692 |
| FA LH CST | rs2650907 | Chr16 : 77359310 | 0.16 | 0.693 |
| FA FMinor | rs2650907 | Chr16 : 77359310 | 0.16 | 0.693 |
| AD RH ATR | rs2650907 | Chr16 : 77359310 | 0.15 | 0.695 |
| FA LH UNC | rs1948719 | Chr16 : 77364184 | 0.15 | 0.697 |
| MD LH CCG | rs2650907 | Chr16 : 77359310 | 0.15 | 0.700 |
| AD RH CAB | rs2650907 | Chr16 : 77359310 | 0.14 | 0.704 |
| FA LH CAB | rs11643211 | Chr16 : 77367649 | 0.14 | 0.706 |
| MD RH ILF | rs3743749 | Chr16 : 77289338 | 0.14 | 0.706 |
| MD LH ILF | rs35478105 | Chr16 : 77291428 | 0.14 | 0.709 |
| FA RH CCG | rs3743749 | Chr16 : 77289338 | 0.14 | 0.711 |
| AD LH CST | rs3743749 | Chr16 : 77289338 | 0.14 | 0.711 |
| FA LH CAB | rs1948719 | Chr16 : 77364184 | 0.14 | 0.712 |
| RD LH CAB | rs35296483 | Chr16 : 77293121 | 0.13 | 0.716 |
| RD LH CAB | rs12935229 | Chr16 : 77294998 | 0.13 | 0.716 |
| RD LH CAB | rs12935394 | Chr16 : 77295093 | 0.13 | 0.716 |
| RD RH SLFP | rs2650907 | Chr16 : 77359310 | 0.13 | 0.720 |
| FA LH CST | rs11643211 | Chr16 : 77367649 | 0.13 | 0.720 |
| RD RH CCG | rs2650907 | Chr16 : 77359310 | 0.13 | 0.720 |
| FA LH ATR | rs11643211 | Chr16 : 77367649 | 0.13 | 0.720 |
| MD RH ATR | rs2650907 | Chr16 : 77359310 | 0.13 | 0.722 |
| AD RH SLFT | rs3743749 | Chr16 : 77289338 | 0.13 | 0.723 |
| AD RH SLFT | rs35296483 | Chr16 : 77293121 | 0.12 | 0.726 |
| AD RH SLFT | rs12935229 | Chr16 : 77294998 | 0.12 | 0.726 |
| AD RH SLFT | rs12935394 | Chr16 : 77295093 | 0.12 | 0.726 |
| FA FMinor | rs35478105 | Chr16 : 77291428 | 0.12 | 0.726 |
| MD FMajor | rs3743749 | Chr16 : 77289338 | 0.12 | 0.727 |
| MD LH CAB | rs1948719 | Chr16 : 77364184 | 0.12 | 0.730 |
| AD LH SLFP | rs2650907 | Chr16 : 77359310 | 0.11 | 0.737 |
| MD RH CCG | rs2650907 | Chr16 : 77359310 | 0.11 | 0.741 |
| AD RH UNC | rs3743749 | Chr16 : 77289338 | 0.11 | 0.744 |
| AD LH CAB | rs3743749 | Chr16 : 77289338 | 0.10 | 0.748 |
| AD LH ATR | rs3743749 | Chr16 : 77289338 | 0.10 | 0.753 |
| AD RH CST | rs2650907 | Chr16 : 77359310 | 0.10 | 0.756 |
| FA LH CCG | rs2650907 | Chr16 : 77359310 | 0.09 | 0.762 |
| MD RH SLFP | rs1948719 | Chr16 : 77364184 | 0.09 | 0.766 |
| AD LH CCG | rs3743749 | Chr16 : 77289338 | 0.09 | 0.766 |
| RD RH ATR | rs1948719 | Chr16 : 77364184 | 0.09 | 0.767 |
| MD LH CST | rs2650907 | Chr16 : 77359310 | 0.08 | 0.772 |
| AD RH SLFT | rs2650907 | Chr16 : 77359310 | 0.08 | 0.772 |
| AD LH SLFP | rs35478105 | Chr16 : 77291428 | 0.08 | 0.773 |
| AD LH ATR | rs35478105 | Chr16 : 77291428 | 0.08 | 0.773 |
| MD FMajor | rs1948719 | Chr16 : 77364184 | 0.08 | 0.775 |
| MD RH CST | rs1948719 | Chr16 : 77364184 | 0.08 | 0.776 |
| RD RH ATR | rs2650907 | Chr16 : 77359310 | 0.08 | 0.784 |
| MD LH ATR | rs2650907 | Chr16 : 77359310 | 0.07 | 0.786 |
| RD RH ILF | rs3743749 | Chr16 : 77289338 | 0.07 | 0.787 |
| AD RH ATR | rs3743749 | Chr16 : 77289338 | 0.07 | 0.787 |
| MD FMinor | rs35478105 | Chr16 : 77291428 | 0.07 | 0.788 |
| RD RH CST | rs1948719 | Chr16 : 77364184 | 0.07 | 0.790 |
| FA RH UNC | rs2650907 | Chr16 : 77359310 | 0.07 | 0.793 |
| RD RH UNC | rs35478105 | Chr16 : 77291428 | 0.07 | 0.794 |
| FA RH CAB | rs35478105 | Chr16 : 77291428 | 0.07 | 0.794 |
| RD FMajor | rs11643211 | Chr16 : 77367649 | 0.07 | 0.795 |
| AD RH ILF | rs35478105 | Chr16 : 77291428 | 0.07 | 0.796 |
| RD FMinor | rs2650907 | Chr16 : 77359310 | 0.07 | 0.798 |
| AD RH CCG | rs3743749 | Chr16 : 77289338 | 0.06 | 0.803 |
| FA LH SLFP | rs11643211 | Chr16 : 77367649 | 0.06 | 0.807 |
| MD RH CST | rs35478105 | Chr16 : 77291428 | 0.06 | 0.807 |
| RD LH ATR | rs35478105 | Chr16 : 77291428 | 0.06 | 0.809 |
| FA LH SLFT | rs2650907 | Chr16 : 77359310 | 0.06 | 0.813 |
| AD RH ILF | rs2650907 | Chr16 : 77359310 | 0.06 | 0.814 |
| MD LH UNC | rs2650907 | Chr16 : 77359310 | 0.06 | 0.814 |
| AD RH ATR | rs35296483 | Chr16 : 77293121 | 0.05 | 0.816 |
| AD RH ATR | rs12935229 | Chr16 : 77294998 | 0.05 | 0.816 |
| AD RH ATR | rs12935394 | Chr16 : 77295093 | 0.05 | 0.816 |
| FA RH UNC | rs35478105 | Chr16 : 77291428 | 0.05 | 0.822 |
| RD LH UNC | rs11643211 | Chr16 : 77367649 | 0.05 | 0.825 |
| MD FMajor | rs2650907 | Chr16 : 77359310 | 0.05 | 0.825 |
| MD RH SLFP | rs2650907 | Chr16 : 77359310 | 0.05 | 0.825 |
| RD LH CST | rs35478105 | Chr16 : 77291428 | 0.05 | 0.826 |
| AD LH CAB | rs1948719 | Chr16 : 77364184 | 0.05 | 0.826 |
| MD LH UNC | rs35478105 | Chr16 : 77291428 | 0.05 | 0.826 |
| FA LH ILF | rs2650907 | Chr16 : 77359310 | 0.05 | 0.827 |
| MD RH CAB | rs1948719 | Chr16 : 77364184 | 0.05 | 0.831 |
| RD RH CCG | rs1948719 | Chr16 : 77364184 | 0.05 | 0.831 |
| MD LH CST | rs35478105 | Chr16 : 77291428 | 0.05 | 0.832 |
| MD LH CAB | rs11643211 | Chr16 : 77367649 | 0.04 | 0.835 |
| MD RH SLFP | rs35478105 | Chr16 : 77291428 | 0.04 | 0.835 |
| MD LH CST | rs1948719 | Chr16 : 77364184 | 0.04 | 0.838 |
| FA LH CCG | rs3743749 | Chr16 : 77289338 | 0.04 | 0.839 |
| RD LH CCG | rs3743749 | Chr16 : 77289338 | 0.04 | 0.841 |
| FA LH CAB | rs2650907 | Chr16 : 77359310 | 0.04 | 0.843 |
| FA RH CCG | rs35478105 | Chr16 : 77291428 | 0.04 | 0.844 |
| FA FMajor | rs11643211 | Chr16 : 77367649 | 0.04 | 0.845 |
| AD FMajor | rs35478105 | Chr16 : 77291428 | 0.04 | 0.846 |
| AD RH CAB | rs3743749 | Chr16 : 77289338 | 0.04 | 0.847 |
| RD LH CCG | rs1948719 | Chr16 : 77364184 | 0.04 | 0.848 |
| FA RH CST | rs3743749 | Chr16 : 77289338 | 0.04 | 0.851 |
| AD FMinor | rs3743749 | Chr16 : 77289338 | 0.03 | 0.858 |
| AD LH ATR | rs35296483 | Chr16 : 77293121 | 0.03 | 0.860 |
| AD LH ATR | rs12935229 | Chr16 : 77294998 | 0.03 | 0.860 |
| AD LH ATR | rs12935394 | Chr16 : 77295093 | 0.03 | 0.860 |
| RD RH CCG | rs35478105 | Chr16 : 77291428 | 0.03 | 0.860 |
| AD LH ILF | rs2650907 | Chr16 : 77359310 | 0.03 | 0.861 |
| FA RH ATR | rs11643211 | Chr16 : 77367649 | 0.03 | 0.866 |
| RD RH CAB | rs35478105 | Chr16 : 77291428 | 0.02 | 0.879 |
| FA LH CCG | rs35296483 | Chr16 : 77293121 | 0.02 | 0.880 |
| FA LH CCG | rs12935229 | Chr16 : 77294998 | 0.02 | 0.880 |
| FA LH CCG | rs12935394 | Chr16 : 77295093 | 0.02 | 0.880 |
| AD LH CST | rs35478105 | Chr16 : 77291428 | 0.02 | 0.880 |
| RD RH UNC | rs2650907 | Chr16 : 77359310 | 0.02 | 0.891 |
| FA RH CCG | rs2650907 | Chr16 : 77359310 | 0.02 | 0.895 |
| FA FMinor | rs35296483 | Chr16 : 77293121 | 0.02 | 0.901 |
| FA FMinor | rs12935229 | Chr16 : 77294998 | 0.02 | 0.901 |
| FA FMinor | rs12935394 | Chr16 : 77295093 | 0.02 | 0.901 |
| FA RH CST | rs11643211 | Chr16 : 77367649 | 0.01 | 0.903 |
| FA RH ATR | rs2650907 | Chr16 : 77359310 | 0.01 | 0.904 |
| FA RH CCG | rs1948719 | Chr16 : 77364184 | 0.01 | 0.905 |
| AD RH CAB | rs35478105 | Chr16 : 77291428 | 0.01 | 0.908 |
| RD FMinor | rs35478105 | Chr16 : 77291428 | 0.01 | 0.914 |
| RD RH SLFP | rs1948719 | Chr16 : 77364184 | 0.01 | 0.919 |
| RD RH CAB | rs11643211 | Chr16 : 77367649 | 0.01 | 0.924 |
| AD RH CCG | rs11643211 | Chr16 : 77367649 | 0.01 | 0.929 |
| RD LH ILF | rs2650907 | Chr16 : 77359310 | 0.01 | 0.931 |
| MD RH CST | rs2650907 | Chr16 : 77359310 | 0.01 | 0.932 |
| RD FMajor | rs3743749 | Chr16 : 77289338 | 0.01 | 0.936 |
| AD LH SLFT | rs2650907 | Chr16 : 77359310 | 0.01 | 0.936 |
| RD LH CCG | rs2650907 | Chr16 : 77359310 | 0.01 | 0.937 |
| RD FMajor | rs2650907 | Chr16 : 77359310 | 0.01 | 0.941 |
| RD LH UNC | rs35478105 | Chr16 : 77291428 | 0.01 | 0.941 |
| MD LH SLFT | rs2650907 | Chr16 : 77359310 | 0.01 | 0.943 |
| AD RH ATR | rs35478105 | Chr16 : 77291428 | 0.01 | 0.944 |
| AD LH CCG | rs35296483 | Chr16 : 77293121 | 0.00 | 0.945 |
| AD LH CCG | rs12935229 | Chr16 : 77294998 | 0.00 | 0.945 |
| AD LH CCG | rs12935394 | Chr16 : 77295093 | 0.00 | 0.945 |
| AD LH SLFP | rs35296483 | Chr16 : 77293121 | 0.00 | 0.947 |
| AD LH SLFP | rs12935229 | Chr16 : 77294998 | 0.00 | 0.947 |
| AD LH SLFP | rs12935394 | Chr16 : 77295093 | 0.00 | 0.947 |
| RD RH CST | rs2650907 | Chr16 : 77359310 | 0.00 | 0.948 |
| AD RH CST | rs35478105 | Chr16 : 77291428 | 0.00 | 0.948 |
| FA LH SLFP | rs1948719 | Chr16 : 77364184 | 0.00 | 0.949 |
| FA RH ILF | rs3743749 | Chr16 : 77289338 | 0.00 | 0.951 |
| RD LH CST | rs2650907 | Chr16 : 77359310 | 0.00 | 0.953 |
| RD LH SLFT | rs2650907 | Chr16 : 77359310 | 0.00 | 0.955 |
| FA RH ILF | rs1948719 | Chr16 : 77364184 | 0.00 | 0.956 |
| RD LH UNC | rs2650907 | Chr16 : 77359310 | 0.00 | 0.957 |
| MD RH CAB | rs35478105 | Chr16 : 77291428 | 0.00 | 0.957 |
| AD RH SLFT | rs35478105 | Chr16 : 77291428 | 0.00 | 0.958 |
| RD LH CCG | rs35296483 | Chr16 : 77293121 | 0.00 | 0.960 |
| RD LH CCG | rs12935229 | Chr16 : 77294998 | 0.00 | 0.960 |
| RD LH CCG | rs12935394 | Chr16 : 77295093 | 0.00 | 0.960 |
| RD RH CAB | rs1948719 | Chr16 : 77364184 | 0.00 | 0.960 |
| AD LH SLFT | rs35478105 | Chr16 : 77291428 | 0.00 | 0.960 |
| RD LH CAB | rs11643211 | Chr16 : 77367649 | 0.00 | 0.974 |
| RD LH ATR | rs2650907 | Chr16 : 77359310 | 0.00 | 0.976 |
| FA LH CCG | rs35478105 | Chr16 : 77291428 | 0.00 | 0.976 |
| FA LH CAB | rs35478105 | Chr16 : 77291428 | 0.00 | 0.977 |
| MD LH ATR | rs35478105 | Chr16 : 77291428 | 0.00 | 0.978 |
| MD LH ILF | rs2650907 | Chr16 : 77359310 | 0.00 | 0.979 |
| RD RH SLFT | rs1948719 | Chr16 : 77364184 | 0.00 | 0.980 |
| AD RH SLFP | rs2650907 | Chr16 : 77359310 | 0.00 | 0.982 |
| AD LH CCG | rs35478105 | Chr16 : 77291428 | 0.00 | 0.986 |
| MD LH CCG | rs35478105 | Chr16 : 77291428 | 0.00 | 0.989 |
| MD LH CCG | rs3743749 | Chr16 : 77289338 | 0.00 | 0.991 |
| FA LH ILF | rs3743749 | Chr16 : 77289338 | 0.00 | 0.991 |
| AD RH CCG | rs2650907 | Chr16 : 77359310 | 0.00 | 0.993 |
| RD LH CCG | rs35478105 | Chr16 : 77291428 | 0.00 | 0.995 |
| FA RH SLFP | rs11643211 | Chr16 : 77367649 | 0.00 | 0.996 |
| FA FMajor | rs2650907 | Chr16 : 77359310 | 0.00 | 0.999 |
| FA LH CST | rs35478105 | Chr16 : 77291428 | 0.00 | 0.999 |
| MD LH CCG | rs35296483 | Chr16 : 77293121 | 0.00 | 0.999 |
| MD LH CCG | rs12935229 | Chr16 : 77294998 | 0.00 | 0.999 |
| MD LH CCG | rs12935394 | Chr16 : 77295093 | 0.00 | 0.999 |

Each model tested the interaction between genotype (additive or dominant coding) and diagnostic group (major depressive disorder [MDD] vs healthy controls [HC]) on tract-level axial diffusivity (AD), controlling for age and sex. F statistics and nominal p-values are reported, with nominally significant results (p < 0.05, uncorrected) highlighted in boldface. No results survived false discovery rate (FDR) correction. Genomic positions are based on the human reference genome build GRCh38.

Abbreviations: AD, axial diffusivity; ATR, anterior thalamic radiation; CAB, cingulum angular bundle; CCG, cingulum cingulate gyrus bundle; CST, corticospinal tract; FA, fractional anisotropy; FMajor, forceps major; FMinor, forceps minor; HC, healthy controls; ILF, inferior longitudinal fasciculus; LH, left hemisphere; MD, mean diffusivity; MDD, major depressive disorder; RD, radial diffusivity; RH, right hemisphere; SLFP, superior longitudinal fasciculus–parietal bundle; SLFT, superior longitudinal fasciculus–temporal bundle; UNC, uncinate fasciculus.

**Supplementary Table S3. Linear regression results for HDRS × SNP interaction on white matter integrity**

| Region | SNP | Genomic position  (GRCh38) | β | R² | P-value |
| --- | --- | --- | --- | --- | --- |
| MD LH ILF | rs1948719 | Chr16 : 77364184 | 3.73E-06 | 0.06 | **0.001** |
| FA RH SLFT | rs35296483 | Chr16 : 77293121 | -9.69E-04 | 0.08 | **0.002** |
| FA RH SLFT | rs12935229 | Chr16 : 77294998 | -9.69E-04 | 0.08 | **0.002** |
| FA RH SLFT | rs12935394 | Chr16 : 77295093 | -9.69E-04 | 0.08 | **0.002** |
| FA LH ILF | rs35478105 | Chr16 : 77291428 | -1.20E-03 | 0.08 | **0.003** |
| FA RH CCG | rs35296483 | Chr16 : 77293121 | -2.39E-03 | 0.05 | **0.003** |
| FA RH CCG | rs12935229 | Chr16 : 77294998 | -2.39E-03 | 0.05 | **0.003** |
| FA RH CCG | rs12935394 | Chr16 : 77295093 | -2.39E-03 | 0.05 | **0.003** |
| RD LH ILF | rs1948719 | Chr16 : 77364184 | 3.24E-06 | 0.06 | **0.003** |
| AD LH ILF | rs1948719 | Chr16 : 77364184 | 4.70E-06 | 0.05 | **0.003** |
| FA LH SLFT | rs35478105 | Chr16 : 77291428 | -9.60E-04 | 0.06 | **0.005** |
| RD LH SLFT | rs35478105 | Chr16 : 77291428 | 1.64E-06 | 0.09 | **0.005** |
| AD LH CCG | rs1948719 | Chr16 : 77364184 | 5.13E-06 | 0.06 | **0.007** |
| FA RH ATR | rs35296483 | Chr16 : 77293121 | -1.00E-03 | 0.09 | **0.009** |
| FA RH ATR | rs12935394 | Chr16 : 77295093 | -1.00E-03 | 0.09 | **0.009** |
| FA RH ATR | rs12935229 | Chr16 : 77294998 | -1.00E-03 | 0.09 | **0.009** |
| FA RH ILF | rs35296483 | Chr16 : 77293121 | -1.10E-03 | 0.07 | **0.009** |
| FA RH ILF | rs12935394 | Chr16 : 77295093 | -1.10E-03 | 0.07 | **0.009** |
| FA RH ILF | rs12935229 | Chr16 : 77294998 | -1.10E-03 | 0.07 | **0.009** |
| FA LH ATR | rs3743749 | Chr16 : 77289338 | 8.65E-04 | 0.12 | **0.009** |
| AD FMinor | rs1948719 | Chr16 : 77364184 | 4.41E-06 | 0.02 | **0.009** |
| FA RH SLFP | rs35478105 | Chr16 : 77291428 | -9.30E-04 | 0.10 | **0.011** |
| RD RH CCG | rs12935229 | Chr16 : 77294998 | 2.31E-06 | 0.05 | **0.012** |
| RD RH CCG | rs12935394 | Chr16 : 77295093 | 2.31E-06 | 0.05 | **0.012** |
| RD RH CCG | rs35296483 | Chr16 : 77293121 | 2.31E-06 | 0.05 | **0.012** |
| RD LH ILF | rs35478105 | Chr16 : 77291428 | 1.58E-06 | 0.06 | **0.012** |
| MD LH CCG | rs1948719 | Chr16 : 77364184 | 2.99E-06 | 0.06 | **0.013** |
| RD RH ILF | rs12935394 | Chr16 : 77295093 | 1.61E-06 | 0.07 | **0.013** |
| RD RH ILF | rs35296483 | Chr16 : 77293121 | 1.61E-06 | 0.07 | **0.013** |
| RD RH ILF | rs12935229 | Chr16 : 77294998 | 1.61E-06 | 0.07 | **0.013** |
| AD FMajor | rs1948719 | Chr16 : 77364184 | 4.67E-06 | 0.03 | **0.014** |
| AD RH CAB | rs12935394 | Chr16 : 77295093 | 4.81E-06 | 0.04 | **0.015** |
| AD RH CAB | rs35296483 | Chr16 : 77293121 | 4.81E-06 | 0.04 | **0.015** |
| AD RH CAB | rs12935229 | Chr16 : 77294998 | 4.81E-06 | 0.04 | **0.015** |
| AD LH SLFT | rs1948719 | Chr16 : 77364184 | 3.25E-06 | 0.05 | **0.019** |
| FA RH SLFP | rs35296483 | Chr16 : 77293121 | -8.31E-04 | 0.09 | **0.022** |
| FA RH SLFP | rs12935394 | Chr16 : 77295093 | -8.31E-04 | 0.09 | **0.022** |
| FA RH SLFP | rs12935229 | Chr16 : 77294998 | -8.31E-04 | 0.09 | **0.022** |
| AD LH ATR | rs1948719 | Chr16 : 77364184 | 3.21E-06 | 0.05 | **0.022** |
| MD LH SLFT | rs35478105 | Chr16 : 77291428 | 1.39E-06 | 0.08 | **0.023** |
| AD LH UNC | rs1948719 | Chr16 : 77364184 | 3.08E-06 | 0.03 | **0.024** |
| AD LH CAB | rs12935229 | Chr16 : 77294998 | 3.79E-06 | 0.02 | **0.024** |
| AD LH CAB | rs12935394 | Chr16 : 77295093 | 3.79E-06 | 0.02 | **0.024** |
| AD LH CAB | rs35296483 | Chr16 : 77293121 | 3.79E-06 | 0.02 | **0.024** |
| FA RH CST | rs35296483 | Chr16 : 77293121 | -9.61E-04 | 0.11 | **0.027** |
| FA RH CST | rs12935394 | Chr16 : 77295093 | -9.61E-04 | 0.11 | **0.027** |
| FA RH CST | rs12935229 | Chr16 : 77294998 | -9.61E-04 | 0.11 | **0.027** |
| FA LH SLFT | rs12935394 | Chr16 : 77295093 | -7.35E-04 | 0.05 | **0.029** |
| FA LH SLFT | rs12935229 | Chr16 : 77294998 | -7.35E-04 | 0.05 | **0.029** |
| FA LH SLFT | rs35296483 | Chr16 : 77293121 | -7.35E-04 | 0.05 | **0.029** |
| RD RH SLFT | rs35296483 | Chr16 : 77293121 | 1.26E-06 | 0.07 | **0.029** |
| RD RH SLFT | rs12935229 | Chr16 : 77294998 | 1.26E-06 | 0.07 | **0.029** |
| RD RH SLFT | rs12935394 | Chr16 : 77295093 | 1.26E-06 | 0.07 | **0.029** |
| FA LH CCG | rs12935394 | Chr16 : 77295093 | -1.35E-03 | 0.05 | **0.029** |
| FA LH CCG | rs35296483 | Chr16 : 77293121 | -1.35E-03 | 0.05 | **0.029** |
| FA LH CCG | rs12935229 | Chr16 : 77294998 | -1.35E-03 | 0.05 | **0.029** |
| RD RH UNC | rs12935394 | Chr16 : 77295093 | 1.53E-06 | 0.08 | **0.029** |
| RD RH UNC | rs12935229 | Chr16 : 77294998 | 1.53E-06 | 0.08 | **0.029** |
| RD RH UNC | rs35296483 | Chr16 : 77293121 | 1.53E-06 | 0.08 | **0.029** |
| FA LH ATR | rs35296483 | Chr16 : 77293121 | -7.97E-04 | 0.11 | **0.031** |
| FA LH ATR | rs12935394 | Chr16 : 77295093 | -7.97E-04 | 0.11 | **0.031** |
| FA LH ATR | rs12935229 | Chr16 : 77294998 | -7.97E-04 | 0.11 | **0.031** |
| MD LH UNC | rs1948719 | Chr16 : 77364184 | 2.16E-06 | 0.08 | **0.034** |
| MD RH ILF | rs1948719 | Chr16 : 77364184 | 2.55E-06 | 0.06 | **0.034** |
| AD RH ILF | rs1948719 | Chr16 : 77364184 | 3.60E-06 | 0.04 | **0.035** |
| FA RH ILF | rs147053635 | Chr16 : 77284049 | 2.08E-03 | 0.06 | **0.036** |
| MD RH UNC | rs12935394 | Chr16 : 77295093 | 1.47E-06 | 0.06 | **0.042** |
| MD RH UNC | rs12935229 | Chr16 : 77294998 | 1.47E-06 | 0.06 | **0.042** |
| MD RH UNC | rs35296483 | Chr16 : 77293121 | 1.47E-06 | 0.06 | **0.042** |
| AD RH CCG | rs35478105 | Chr16 : 77291428 | 1.73E-06 | 0.02 | **0.045** |
| RD RH SLFP | rs12935229 | Chr16 : 77294998 | 1.29E-06 | 0.08 | **0.046** |
| RD RH SLFP | rs12935394 | Chr16 : 77295093 | 1.29E-06 | 0.08 | **0.046** |
| RD RH SLFP | rs35296483 | Chr16 : 77293121 | 1.29E-06 | 0.08 | **0.046** |
| MD LH ATR | rs1948719 | Chr16 : 77364184 | 2.09E-06 | 0.10 | **0.046** |
| RD LH CST | rs3743750 | Chr16 : 77289313 | -3.77E-06 | 0.09 | **0.046** |
| RD LH CST | rs757155340 | Chr16 : 77284051 | -3.77E-06 | 0.09 | **0.046** |
| FA LH SLFP | rs35478105 | Chr16 : 77291428 | -6.86E-04 | 0.08 | **0.048** |
| MD LH CST | rs3743750 | Chr16 : 77289313 | -3.63E-06 | 0.07 | **0.048** |
| MD LH CST | rs757155340 | Chr16 : 77284051 | -3.63E-06 | 0.07 | **0.048** |
| MD RH CAB | rs12935229 | Chr16 : 77294998 | 3.32E-06 | 0.02 | 0.051 |
| MD RH CAB | rs12935394 | Chr16 : 77295093 | 3.32E-06 | 0.02 | 0.051 |
| MD RH CAB | rs35296483 | Chr16 : 77293121 | 3.32E-06 | 0.02 | 0.051 |
| RD LH SLFP | rs35478105 | Chr16 : 77291428 | 1.17E-06 | 0.11 | 0.051 |
| RD LH ATR | rs12935229 | Chr16 : 77294998 | 1.12E-06 | 0.12 | 0.051 |
| RD LH ATR | rs35296483 | Chr16 : 77293121 | 1.12E-06 | 0.12 | 0.051 |
| RD LH ATR | rs12935394 | Chr16 : 77295093 | 1.12E-06 | 0.12 | 0.051 |
| MD LH SLFT | rs1948719 | Chr16 : 77364184 | 2.07E-06 | 0.07 | 0.052 |
| MD RH ILF | rs35296483 | Chr16 : 77293121 | 1.32E-06 | 0.05 | 0.052 |
| MD RH ILF | rs12935229 | Chr16 : 77294998 | 1.32E-06 | 0.05 | 0.052 |
| MD RH ILF | rs12935394 | Chr16 : 77295093 | 1.32E-06 | 0.05 | 0.052 |
| RD RH ATR | rs12935394 | Chr16 : 77295093 | 1.09E-06 | 0.09 | 0.053 |
| RD RH ATR | rs12935229 | Chr16 : 77294998 | 1.09E-06 | 0.09 | 0.053 |
| RD RH ATR | rs35296483 | Chr16 : 77293121 | 1.09E-06 | 0.09 | 0.053 |
| FA RH ATR | rs3743749 | Chr16 : 77289338 | 6.68E-04 | 0.09 | 0.054 |
| MD LH ILF | rs12935394 | Chr16 : 77295093 | 1.24E-06 | 0.04 | 0.054 |
| MD LH ILF | rs35296483 | Chr16 : 77293121 | 1.24E-06 | 0.04 | 0.054 |
| MD LH ILF | rs12935229 | Chr16 : 77294998 | 1.24E-06 | 0.04 | 0.054 |
| MD RH UNC | rs1948719 | Chr16 : 77364184 | 2.46E-06 | 0.06 | 0.054 |
| MD FMinor | rs1948719 | Chr16 : 77364184 | 2.02E-06 | 0.05 | 0.054 |
| FA LH CST | rs3743749 | Chr16 : 77289338 | 7.39E-04 | 0.10 | 0.056 |
| RD LH CCG | rs12935229 | Chr16 : 77294998 | 1.42E-06 | 0.05 | 0.056 |
| RD LH CCG | rs35296483 | Chr16 : 77293121 | 1.42E-06 | 0.05 | 0.056 |
| RD LH CCG | rs12935394 | Chr16 : 77295093 | 1.42E-06 | 0.05 | 0.056 |
| AD RH CCG | rs12935394 | Chr16 : 77295093 | -1.61E-06 | 0.03 | 0.056 |
| AD RH CCG | rs12935229 | Chr16 : 77294998 | -1.61E-06 | 0.03 | 0.056 |
| AD RH CCG | rs35296483 | Chr16 : 77293121 | -1.61E-06 | 0.03 | 0.056 |
| AD LH CST | rs1948719 | Chr16 : 77364184 | 2.76E-06 | 0.04 | 0.058 |
| AD RH UNC | rs1948719 | Chr16 : 77364184 | 3.20E-06 | 0.03 | 0.060 |
| MD RH SLFP | rs3743750 | Chr16 : 77289313 | -3.42E-06 | 0.07 | 0.062 |
| MD RH SLFP | rs757155340 | Chr16 : 77284051 | -3.42E-06 | 0.07 | 0.062 |
| FA RH UNC | rs12935229 | Chr16 : 77294998 | -8.08E-04 | 0.08 | 0.064 |
| FA RH UNC | rs35296483 | Chr16 : 77293121 | -8.08E-04 | 0.08 | 0.064 |
| FA RH UNC | rs12935394 | Chr16 : 77295093 | -8.08E-04 | 0.08 | 0.064 |
| FA LH CST | rs3743750 | Chr16 : 77289313 | 2.16E-03 | 0.10 | 0.066 |
| FA LH CST | rs757155340 | Chr16 : 77284051 | 2.16E-03 | 0.10 | 0.066 |
| FA LH SLFP | rs12935394 | Chr16 : 77295093 | -6.26E-04 | 0.07 | 0.066 |
| FA LH SLFP | rs35296483 | Chr16 : 77293121 | -6.26E-04 | 0.07 | 0.066 |
| FA LH SLFP | rs12935229 | Chr16 : 77294998 | -6.26E-04 | 0.07 | 0.066 |
| AD RH CST | rs1948719 | Chr16 : 77364184 | 3.11E-06 | 0.04 | 0.068 |
| RD LH ILF | rs35296483 | Chr16 : 77293121 | 1.13E-06 | 0.05 | 0.068 |
| RD LH ILF | rs12935229 | Chr16 : 77294998 | 1.13E-06 | 0.05 | 0.068 |
| RD LH ILF | rs12935394 | Chr16 : 77295093 | 1.13E-06 | 0.05 | 0.068 |
| AD RH SLFP | rs3743750 | Chr16 : 77289313 | -4.13E-06 | 0.04 | 0.070 |
| AD RH SLFP | rs757155340 | Chr16 : 77284051 | -4.13E-06 | 0.04 | 0.070 |
| MD RH CCG | rs1948719 | Chr16 : 77364184 | 2.10E-06 | 0.05 | 0.070 |
| FA RH SLFP | rs147053635 | Chr16 : 77284049 | 1.54E-03 | 0.08 | 0.071 |
| MD RH ATR | rs1948719 | Chr16 : 77364184 | 1.87E-06 | 0.07 | 0.072 |
| RD LH ATR | rs757155340 | Chr16 : 77284051 | -2.83E-06 | 0.12 | 0.073 |
| RD LH ATR | rs3743750 | Chr16 : 77289313 | -2.83E-06 | 0.12 | 0.073 |
| FA LH CST | rs35296483 | Chr16 : 77293121 | -7.59E-04 | 0.11 | 0.076 |
| FA LH CST | rs12935229 | Chr16 : 77294998 | -7.59E-04 | 0.11 | 0.076 |
| FA LH CST | rs12935394 | Chr16 : 77295093 | -7.59E-04 | 0.11 | 0.076 |
| MD RH SLFT | rs3743750 | Chr16 : 77289313 | -3.13E-06 | 0.06 | 0.076 |
| MD RH SLFT | rs757155340 | Chr16 : 77284051 | -3.13E-06 | 0.06 | 0.076 |
| RD RH ILF | rs1948719 | Chr16 : 77364184 | 2.03E-06 | 0.06 | 0.077 |
| RD LH UNC | rs12935394 | Chr16 : 77295093 | 1.04E-06 | 0.10 | 0.078 |
| RD LH UNC | rs35296483 | Chr16 : 77293121 | 1.04E-06 | 0.10 | 0.078 |
| RD LH UNC | rs12935229 | Chr16 : 77294998 | 1.04E-06 | 0.10 | 0.078 |
| RD RH ILF | rs147053635 | Chr16 : 77284049 | -2.68E-06 | 0.06 | 0.079 |
| AD RH CST | rs3743750 | Chr16 : 77289313 | -4.62E-06 | 0.04 | 0.080 |
| AD RH CST | rs757155340 | Chr16 : 77284051 | -4.62E-06 | 0.04 | 0.080 |
| FA RH SLFT | rs35478105 | Chr16 : 77291428 | -5.66E-04 | 0.06 | 0.082 |
| MD LH CAB | rs12935394 | Chr16 : 77295093 | 2.43E-06 | 0.03 | 0.082 |
| MD LH CAB | rs35296483 | Chr16 : 77293121 | 2.43E-06 | 0.03 | 0.082 |
| MD LH CAB | rs12935229 | Chr16 : 77294998 | 2.43E-06 | 0.03 | 0.082 |
| RD RH SLFP | rs757155340 | Chr16 : 77284051 | -3.07E-06 | 0.08 | 0.082 |
| RD RH SLFP | rs3743750 | Chr16 : 77289313 | -3.07E-06 | 0.08 | 0.082 |
| AD LH UNC | rs11643211 | Chr16 : 77367648 | -1.49E-06 | 0.02 | 0.083 |
| RD RH CST | rs12935229 | Chr16 : 77294998 | 1.32E-06 | 0.08 | 0.083 |
| RD RH CST | rs35296483 | Chr16 : 77293121 | 1.32E-06 | 0.08 | 0.083 |
| RD RH CST | rs12935394 | Chr16 : 77295093 | 1.32E-06 | 0.08 | 0.083 |
| RD RH ATR | rs3743749 | Chr16 : 77289338 | -8.78E-07 | 0.10 | 0.083 |
| RD RH ATR | rs1948719 | Chr16 : 77364184 | 1.72E-06 | 0.09 | 0.084 |
| RD LH SLFT | rs12935229 | Chr16 : 77294998 | 9.88E-07 | 0.07 | 0.089 |
| RD LH SLFT | rs35296483 | Chr16 : 77293121 | 9.88E-07 | 0.07 | 0.089 |
| RD LH SLFT | rs12935394 | Chr16 : 77295093 | 9.88E-07 | 0.07 | 0.089 |
| RD RH UNC | rs1948719 | Chr16 : 77364184 | 2.10E-06 | 0.07 | 0.090 |
| MD RH CST | rs3743750 | Chr16 : 77289313 | -3.62E-06 | 0.06 | 0.090 |
| MD RH CST | rs757155340 | Chr16 : 77284051 | -3.62E-06 | 0.06 | 0.090 |
| RD LH CST | rs12935394 | Chr16 : 77295093 | 1.17E-06 | 0.09 | 0.090 |
| RD LH CST | rs12935229 | Chr16 : 77294998 | 1.17E-06 | 0.09 | 0.090 |
| RD LH CST | rs35296483 | Chr16 : 77293121 | 1.17E-06 | 0.09 | 0.090 |
| RD RH SLFT | rs757155340 | Chr16 : 77284051 | -2.64E-06 | 0.07 | 0.095 |
| RD RH SLFT | rs3743750 | Chr16 : 77289313 | -2.64E-06 | 0.07 | 0.095 |
| AD RH SLFT | rs3743750 | Chr16 : 77289313 | -4.11E-06 | 0.04 | 0.095 |
| AD RH SLFT | rs757155340 | Chr16 : 77284051 | -4.11E-06 | 0.04 | 0.095 |
| RD LH ATR | rs3743749 | Chr16 : 77289338 | -8.62E-07 | 0.13 | 0.095 |
| MD RH SLFP | rs12935229 | Chr16 : 77294998 | 1.12E-06 | 0.06 | 0.096 |
| MD RH SLFP | rs35296483 | Chr16 : 77293121 | 1.12E-06 | 0.06 | 0.096 |
| MD RH SLFP | rs12935394 | Chr16 : 77295093 | 1.12E-06 | 0.06 | 0.096 |
| MD LH SLFP | rs35478105 | Chr16 : 77291428 | 1.04E-06 | 0.09 | 0.098 |
| FA LH UNC | rs12935229 | Chr16 : 77294998 | -6.91E-04 | 0.10 | 0.099 |
| FA LH UNC | rs12935394 | Chr16 : 77295093 | -6.91E-04 | 0.10 | 0.099 |
| FA LH UNC | rs35296483 | Chr16 : 77293121 | -6.91E-04 | 0.10 | 0.099 |
| RD RH SLFP | rs35478105 | Chr16 : 77291428 | 1.08E-06 | 0.08 | 0.100 |
| MD FMinor | rs35478105 | Chr16 : 77291428 | 9.94E-07 | 0.05 | 0.101 |
| FA RH SLFT | rs2650907 | Chr16 : 77359310 | -4.67E-04 | 0.06 | 0.101 |
| FA RH ILF | rs2650907 | Chr16 : 77359310 | -6.16E-04 | 0.06 | 0.102 |
| AD LH ILF | rs12935394 | Chr16 : 77295093 | 1.47E-06 | 0.03 | 0.103 |
| AD LH ILF | rs12935229 | Chr16 : 77294998 | 1.47E-06 | 0.03 | 0.103 |
| AD LH ILF | rs35296483 | Chr16 : 77293121 | 1.47E-06 | 0.03 | 0.103 |
| MD RH CAB | rs3743750 | Chr16 : 77289313 | -7.54E-06 | 0.02 | 0.106 |
| MD RH CAB | rs757155340 | Chr16 : 77284051 | -7.54E-06 | 0.02 | 0.106 |
| RD LH UNC | rs1948719 | Chr16 : 77364184 | 1.69E-06 | 0.09 | 0.106 |
| MD RH ILF | rs3743750 | Chr16 : 77289313 | -3.01E-06 | 0.05 | 0.107 |
| MD RH ILF | rs757155340 | Chr16 : 77284051 | -3.01E-06 | 0.05 | 0.107 |
| MD LH ILF | rs35478105 | Chr16 : 77291428 | 1.05E-06 | 0.04 | 0.111 |
| RD RH SLFT | rs147053635 | Chr16 : 77284049 | -2.15E-06 | 0.07 | 0.111 |
| RD RH SLFP | rs3743749 | Chr16 : 77289338 | -9.21E-07 | 0.08 | 0.112 |
| AD FMinor | rs757155340 | Chr16 : 77284051 | -4.19E-06 | 0.01 | 0.112 |
| AD FMinor | rs3743750 | Chr16 : 77289313 | -4.19E-06 | 0.01 | 0.112 |
| AD LH ILF | rs11643211 | Chr16 : 77367648 | -1.59E-06 | 0.03 | 0.113 |
| RD LH UNC | rs3743749 | Chr16 : 77289338 | -8.37E-07 | 0.10 | 0.115 |
| FA RH SLFT | rs147053635 | Chr16 : 77284049 | 1.18E-03 | 0.06 | 0.116 |
| MD LH UNC | rs3743750 | Chr16 : 77289313 | -2.48E-06 | 0.07 | 0.116 |
| MD LH UNC | rs757155340 | Chr16 : 77284051 | -2.48E-06 | 0.07 | 0.116 |
| RD RH CAB | rs757155340 | Chr16 : 77284051 | -7.14E-06 | 0.01 | 0.117 |
| RD RH CAB | rs3743750 | Chr16 : 77289313 | -7.14E-06 | 0.01 | 0.117 |
| MD LH ATR | rs757155340 | Chr16 : 77284051 | -2.53E-06 | 0.10 | 0.120 |
| MD LH ATR | rs3743750 | Chr16 : 77289313 | -2.53E-06 | 0.10 | 0.120 |
| RD RH SLFT | rs35478105 | Chr16 : 77291428 | 9.11E-07 | 0.07 | 0.120 |
| RD RH CAB | rs35296483 | Chr16 : 77293121 | 2.58E-06 | 0.01 | 0.121 |
| RD RH CAB | rs12935394 | Chr16 : 77295093 | 2.58E-06 | 0.01 | 0.121 |
| RD RH CAB | rs12935229 | Chr16 : 77294998 | 2.58E-06 | 0.01 | 0.121 |
| FA LH ATR | rs3743750 | Chr16 : 77289313 | 1.57E-03 | 0.10 | 0.121 |
| FA LH ATR | rs757155340 | Chr16 : 77284051 | 1.57E-03 | 0.10 | 0.121 |
| RD LH SLFP | rs35296483 | Chr16 : 77293121 | 9.07E-07 | 0.10 | 0.123 |
| RD LH SLFP | rs12935229 | Chr16 : 77294998 | 9.07E-07 | 0.10 | 0.123 |
| RD LH SLFP | rs12935394 | Chr16 : 77295093 | 9.07E-07 | 0.10 | 0.123 |
| MD LH ILF | rs11643211 | Chr16 : 77367648 | -1.11E-06 | 0.04 | 0.124 |
| MD LH CST | rs12935394 | Chr16 : 77295093 | 1.03E-06 | 0.07 | 0.124 |
| MD LH CST | rs12935229 | Chr16 : 77294998 | 1.03E-06 | 0.07 | 0.124 |
| MD LH CST | rs35296483 | Chr16 : 77293121 | 1.03E-06 | 0.07 | 0.124 |
| FA LH UNC | rs3743749 | Chr16 : 77289338 | 5.77E-04 | 0.11 | 0.124 |
| MD LH ILF | rs3743750 | Chr16 : 77289313 | -2.71E-06 | 0.04 | 0.125 |
| MD LH ILF | rs757155340 | Chr16 : 77284051 | -2.71E-06 | 0.04 | 0.125 |
| RD RH ILF | rs757155340 | Chr16 : 77284051 | -2.72E-06 | 0.06 | 0.126 |
| RD RH ILF | rs3743750 | Chr16 : 77289313 | -2.72E-06 | 0.06 | 0.126 |
| AD RH CAB | rs3743750 | Chr16 : 77289313 | -8.35E-06 | 0.02 | 0.126 |
| AD RH CAB | rs757155340 | Chr16 : 77284051 | -8.35E-06 | 0.02 | 0.126 |
| MD RH CCG | rs12935394 | Chr16 : 77295093 | 1.01E-06 | 0.04 | 0.127 |
| MD RH CCG | rs35296483 | Chr16 : 77293121 | 1.01E-06 | 0.04 | 0.127 |
| MD RH CCG | rs12935229 | Chr16 : 77294998 | 1.01E-06 | 0.04 | 0.127 |
| MD LH UNC | rs12935229 | Chr16 : 77294998 | 8.80E-07 | 0.07 | 0.127 |
| MD LH UNC | rs12935394 | Chr16 : 77295093 | 8.80E-07 | 0.07 | 0.127 |
| MD LH UNC | rs35296483 | Chr16 : 77293121 | 8.80E-07 | 0.07 | 0.127 |
| AD LH SLFT | rs11643211 | Chr16 : 77367648 | -1.33E-06 | 0.04 | 0.128 |
| RD FMinor | rs35478105 | Chr16 : 77291428 | 9.35E-07 | 0.08 | 0.128 |
| FA RH UNC | rs3743749 | Chr16 : 77289338 | 5.98E-04 | 0.08 | 0.128 |
| MD RH SLFT | rs12935394 | Chr16 : 77295093 | 9.77E-07 | 0.05 | 0.130 |
| MD RH SLFT | rs35296483 | Chr16 : 77293121 | 9.77E-07 | 0.05 | 0.130 |
| MD RH SLFT | rs12935229 | Chr16 : 77294998 | 9.77E-07 | 0.05 | 0.130 |
| RD LH ATR | rs1948719 | Chr16 : 77364184 | 1.53E-06 | 0.12 | 0.132 |
| AD RH ATR | rs1948719 | Chr16 : 77364184 | 2.18E-06 | 0.03 | 0.133 |
| RD RH CST | rs3743750 | Chr16 : 77289313 | -3.12E-06 | 0.07 | 0.134 |
| RD RH CST | rs757155340 | Chr16 : 77284051 | -3.12E-06 | 0.07 | 0.134 |
| FA RH ILF | rs35478105 | Chr16 : 77291428 | -6.43E-04 | 0.06 | 0.136 |
| AD LH ILF | rs757155340 | Chr16 : 77284051 | -3.69E-06 | 0.03 | 0.136 |
| AD LH ILF | rs3743750 | Chr16 : 77289313 | -3.69E-06 | 0.03 | 0.136 |
| AD RH SLFP | rs11643211 | Chr16 : 77367648 | -1.37E-06 | 0.04 | 0.138 |
| AD LH CST | rs757155340 | Chr16 : 77284051 | -3.34E-06 | 0.04 | 0.138 |
| AD LH CST | rs3743750 | Chr16 : 77289313 | -3.34E-06 | 0.04 | 0.138 |
| RD RH ATR | rs3743750 | Chr16 : 77289313 | -2.29E-06 | 0.09 | 0.140 |
| RD RH ATR | rs757155340 | Chr16 : 77284051 | -2.29E-06 | 0.09 | 0.140 |
| FA LH CAB | rs3743749 | Chr16 : 77289338 | 8.36E-04 | 0.05 | 0.142 |
| MD LH SLFP | rs1948719 | Chr16 : 77364184 | 1.60E-06 | 0.08 | 0.143 |
| AD RH CAB | rs1948719 | Chr16 : 77364184 | 5.15E-06 | 0.02 | 0.143 |
| RD LH CCG | rs1948719 | Chr16 : 77364184 | 1.92E-06 | 0.05 | 0.144 |
| MD FMinor | rs757155340 | Chr16 : 77284051 | -2.38E-06 | 0.04 | 0.144 |
| MD FMinor | rs3743750 | Chr16 : 77289313 | -2.38E-06 | 0.04 | 0.144 |
| RD RH ATR | rs35478105 | Chr16 : 77291428 | 8.39E-07 | 0.09 | 0.145 |
| RD RH CAB | rs3743749 | Chr16 : 77289338 | -2.17E-06 | 0.01 | 0.146 |
| AD RH CCG | rs1948719 | Chr16 : 77364184 | 2.16E-06 | 0.02 | 0.147 |
| AD LH SLFT | rs757155340 | Chr16 : 77284051 | -3.10E-06 | 0.04 | 0.149 |
| AD LH SLFT | rs3743750 | Chr16 : 77289313 | -3.10E-06 | 0.04 | 0.149 |
| FA RH SLFP | rs3743749 | Chr16 : 77289338 | 4.71E-04 | 0.08 | 0.149 |
| RD LH SLFT | rs1948719 | Chr16 : 77364184 | 1.48E-06 | 0.07 | 0.149 |
| RD RH UNC | rs3743750 | Chr16 : 77289313 | -2.75E-06 | 0.07 | 0.152 |
| RD RH UNC | rs757155340 | Chr16 : 77284051 | -2.75E-06 | 0.07 | 0.152 |
| RD LH UNC | rs3743750 | Chr16 : 77289313 | -2.32E-06 | 0.09 | 0.153 |
| RD LH UNC | rs757155340 | Chr16 : 77284051 | -2.32E-06 | 0.09 | 0.153 |
| RD RH UNC | rs147053635 | Chr16 : 77284049 | -2.35E-06 | 0.07 | 0.153 |
| AD RH UNC | rs12935394 | Chr16 : 77295093 | 1.36E-06 | 0.02 | 0.158 |
| AD RH UNC | rs35296483 | Chr16 : 77293121 | 1.36E-06 | 0.02 | 0.158 |
| AD RH UNC | rs12935229 | Chr16 : 77294998 | 1.36E-06 | 0.02 | 0.158 |
| RD RH SLFP | rs1948719 | Chr16 : 77364184 | 1.60E-06 | 0.08 | 0.160 |
| MD LH ATR | rs12935229 | Chr16 : 77294998 | 8.32E-07 | 0.10 | 0.161 |
| MD LH ATR | rs35296483 | Chr16 : 77293121 | 8.32E-07 | 0.10 | 0.161 |
| MD LH ATR | rs12935394 | Chr16 : 77295093 | 8.32E-07 | 0.10 | 0.161 |
| MD LH SLFT | rs757155340 | Chr16 : 77284051 | -2.32E-06 | 0.07 | 0.162 |
| MD LH SLFT | rs3743750 | Chr16 : 77289313 | -2.32E-06 | 0.07 | 0.162 |
| RD LH CST | rs3743749 | Chr16 : 77289338 | -8.66E-07 | 0.09 | 0.164 |
| AD LH SLFP | rs3743750 | Chr16 : 77289313 | -3.10E-06 | 0.04 | 0.165 |
| AD LH SLFP | rs757155340 | Chr16 : 77284051 | -3.10E-06 | 0.04 | 0.165 |
| MD RH SLFT | rs147053635 | Chr16 : 77284049 | -2.10E-06 | 0.05 | 0.165 |
| RD LH SLFP | rs1948719 | Chr16 : 77364184 | 1.44E-06 | 0.10 | 0.165 |
| MD RH ATR | rs3743750 | Chr16 : 77289313 | -2.25E-06 | 0.07 | 0.165 |
| MD RH ATR | rs757155340 | Chr16 : 77284051 | -2.25E-06 | 0.07 | 0.165 |
| MD RH CST | rs12935394 | Chr16 : 77295093 | 1.08E-06 | 0.06 | 0.166 |
| MD RH CST | rs35296483 | Chr16 : 77293121 | 1.08E-06 | 0.06 | 0.166 |
| MD RH CST | rs12935229 | Chr16 : 77294998 | 1.08E-06 | 0.06 | 0.166 |
| MD RH CAB | rs3743749 | Chr16 : 77289338 | -2.12E-06 | 0.02 | 0.167 |
| MD RH CST | rs1948719 | Chr16 : 77364184 | 1.90E-06 | 0.06 | 0.169 |
| RD RH CAB | rs35478105 | Chr16 : 77291428 | 2.33E-06 | 0.01 | 0.169 |
| FA RH CAB | rs35478105 | Chr16 : 77291428 | -9.73E-04 | 0.02 | 0.172 |
| RD RH SLFP | rs147053635 | Chr16 : 77284049 | -2.06E-06 | 0.08 | 0.173 |
| FA LH SLFT | rs147053635 | Chr16 : 77284049 | 1.07E-03 | 0.05 | 0.175 |
| AD RH ILF | rs3743750 | Chr16 : 77289313 | -3.59E-06 | 0.03 | 0.177 |
| AD RH ILF | rs757155340 | Chr16 : 77284051 | -3.59E-06 | 0.03 | 0.177 |
| AD LH SLFP | rs1948719 | Chr16 : 77364184 | 1.92E-06 | 0.04 | 0.181 |
| MD RH SLFP | rs3743749 | Chr16 : 77289338 | -8.06E-07 | 0.07 | 0.181 |
| MD RH SLFP | rs1948719 | Chr16 : 77364184 | 1.58E-06 | 0.06 | 0.183 |
| MD FMajor | rs1948719 | Chr16 : 77364184 | 2.07E-06 | 0.02 | 0.183 |
| AD LH UNC | rs757155340 | Chr16 : 77284051 | -2.81E-06 | 0.02 | 0.183 |
| AD LH UNC | rs3743750 | Chr16 : 77289313 | -2.81E-06 | 0.02 | 0.183 |
| FA LH ILF | rs12935394 | Chr16 : 77295093 | -5.19E-04 | 0.06 | 0.190 |
| FA LH ILF | rs35296483 | Chr16 : 77293121 | -5.19E-04 | 0.06 | 0.190 |
| FA LH ILF | rs12935229 | Chr16 : 77294998 | -5.19E-04 | 0.06 | 0.190 |
| RD LH ILF | rs757155340 | Chr16 : 77284051 | -2.23E-06 | 0.05 | 0.191 |
| RD LH ILF | rs3743750 | Chr16 : 77289313 | -2.23E-06 | 0.05 | 0.191 |
| RD LH CST | rs35478105 | Chr16 : 77291428 | 9.20E-07 | 0.08 | 0.192 |
| RD LH SLFT | rs147053635 | Chr16 : 77284049 | -1.78E-06 | 0.07 | 0.192 |
| FA LH SLFP | rs11643211 | Chr16 : 77367648 | -4.93E-04 | 0.08 | 0.193 |
| MD LH SLFP | rs3743750 | Chr16 : 77289313 | -2.20E-06 | 0.08 | 0.195 |
| MD LH SLFP | rs757155340 | Chr16 : 77284051 | -2.20E-06 | 0.08 | 0.195 |
| MD RH UNC | rs757155340 | Chr16 : 77284051 | -2.58E-06 | 0.05 | 0.195 |
| MD RH UNC | rs3743750 | Chr16 : 77289313 | -2.58E-06 | 0.05 | 0.195 |
| RD LH CAB | rs35296483 | Chr16 : 77293121 | 1.76E-06 | 0.03 | 0.196 |
| RD LH CAB | rs12935229 | Chr16 : 77294998 | 1.76E-06 | 0.03 | 0.196 |
| RD LH CAB | rs12935394 | Chr16 : 77295093 | 1.76E-06 | 0.03 | 0.196 |
| FA RH CAB | rs147053635 | Chr16 : 77284049 | 2.11E-03 | 0.02 | 0.198 |
| FA RH UNC | rs147053635 | Chr16 : 77284049 | 1.32E-03 | 0.08 | 0.198 |
| FA LH CST | rs147053635 | Chr16 : 77284049 | 1.29E-03 | 0.10 | 0.200 |
| MD LH SLFT | rs12935394 | Chr16 : 77295093 | 7.74E-07 | 0.06 | 0.200 |
| MD LH SLFT | rs12935229 | Chr16 : 77294998 | 7.74E-07 | 0.06 | 0.200 |
| MD LH SLFT | rs35296483 | Chr16 : 77293121 | 7.74E-07 | 0.06 | 0.200 |
| RD RH CCG | rs1948719 | Chr16 : 77364184 | 2.07E-06 | 0.04 | 0.203 |
| RD RH CCG | rs3743750 | Chr16 : 77289313 | -3.19E-06 | 0.04 | 0.206 |
| RD RH CCG | rs757155340 | Chr16 : 77284051 | -3.19E-06 | 0.04 | 0.206 |
| MD RH ILF | rs147053635 | Chr16 : 77284049 | -2.01E-06 | 0.05 | 0.210 |
| FA LH CAB | rs147053635 | Chr16 : 77284049 | 1.86E-03 | 0.04 | 0.211 |
| RD LH ILF | rs11643211 | Chr16 : 77367648 | -8.63E-07 | 0.05 | 0.211 |
| MD RH UNC | rs147053635 | Chr16 : 77284049 | -2.12E-06 | 0.06 | 0.213 |
| RD RH ILF | rs35478105 | Chr16 : 77291428 | 8.21E-07 | 0.06 | 0.216 |
| MD LH UNC | rs3743749 | Chr16 : 77289338 | -6.33E-07 | 0.07 | 0.221 |
| AD FMinor | rs2650907 | Chr16 : 77359310 | 1.05E-06 | 0.01 | 0.223 |
| FA RH CST | rs3743750 | Chr16 : 77289313 | 1.44E-03 | 0.10 | 0.227 |
| FA RH CST | rs757155340 | Chr16 : 77284051 | 1.44E-03 | 0.10 | 0.227 |
| RD LH SLFT | rs3743750 | Chr16 : 77289313 | -1.92E-06 | 0.07 | 0.228 |
| RD LH SLFT | rs757155340 | Chr16 : 77284051 | -1.92E-06 | 0.07 | 0.228 |
| FA FMajor | rs2650907 | Chr16 : 77359310 | 5.74E-04 | 0.05 | 0.231 |
| MD RH ATR | rs35296483 | Chr16 : 77293121 | 7.06E-07 | 0.06 | 0.232 |
| MD RH ATR | rs12935394 | Chr16 : 77295093 | 7.06E-07 | 0.06 | 0.232 |
| MD RH ATR | rs12935229 | Chr16 : 77294998 | 7.06E-07 | 0.06 | 0.232 |
| MD LH SLFP | rs12935394 | Chr16 : 77295093 | 7.39E-07 | 0.08 | 0.233 |
| MD LH SLFP | rs35296483 | Chr16 : 77293121 | 7.39E-07 | 0.08 | 0.233 |
| MD LH SLFP | rs12935229 | Chr16 : 77294998 | 7.39E-07 | 0.08 | 0.233 |
| MD LH SLFT | rs147053635 | Chr16 : 77284049 | -1.69E-06 | 0.06 | 0.233 |
| MD LH CAB | rs1948719 | Chr16 : 77364184 | 2.93E-06 | 0.02 | 0.236 |
| AD FMajor | rs35478105 | Chr16 : 77291428 | 1.30E-06 | 0.02 | 0.237 |
| AD LH SLFP | rs11643211 | Chr16 : 77367648 | -1.07E-06 | 0.04 | 0.237 |
| RD RH ILF | rs2650907 | Chr16 : 77359310 | 6.84E-07 | 0.06 | 0.239 |
| MD RH ATR | rs3743749 | Chr16 : 77289338 | -6.23E-07 | 0.07 | 0.240 |
| MD LH ATR | rs35478105 | Chr16 : 77291428 | 7.10E-07 | 0.10 | 0.240 |
| AD LH CAB | rs1948719 | Chr16 : 77364184 | 3.49E-06 | 0.01 | 0.241 |
| MD RH ATR | rs35478105 | Chr16 : 77291428 | 7.02E-07 | 0.07 | 0.244 |
| MD RH SLFT | rs1948719 | Chr16 : 77364184 | 1.33E-06 | 0.05 | 0.244 |
| MD LH SLFP | rs3743749 | Chr16 : 77289338 | -6.41E-07 | 0.09 | 0.249 |
| MD LH CST | rs35478105 | Chr16 : 77291428 | 7.88E-07 | 0.07 | 0.250 |
| FA RH UNC | rs3743750 | Chr16 : 77289313 | 1.38E-03 | 0.08 | 0.250 |
| FA RH UNC | rs757155340 | Chr16 : 77284051 | 1.38E-03 | 0.08 | 0.250 |
| AD RH ATR | rs147053635 | Chr16 : 77284049 | -2.22E-06 | 0.02 | 0.250 |
| MD RH CAB | rs35478105 | Chr16 : 77291428 | 1.99E-06 | 0.01 | 0.251 |
| FA LH CST | rs35478105 | Chr16 : 77291428 | -5.03E-04 | 0.09 | 0.251 |
| AD FMinor | rs35478105 | Chr16 : 77291428 | 1.11E-06 | 0.00 | 0.258 |
| MD RH CAB | rs1948719 | Chr16 : 77364184 | 3.40E-06 | 0.02 | 0.258 |
| FA RH SLFP | rs1948719 | Chr16 : 77364184 | -7.21E-04 | 0.08 | 0.260 |
| AD LH SLFT | rs35478105 | Chr16 : 77291428 | 8.99E-07 | 0.04 | 0.260 |
| FA RH ATR | rs35478105 | Chr16 : 77291428 | -4.41E-04 | 0.08 | 0.261 |
| RD LH ILF | rs147053635 | Chr16 : 77284049 | -1.64E-06 | 0.04 | 0.261 |
| AD RH CAB | rs3743749 | Chr16 : 77289338 | -2.01E-06 | 0.02 | 0.264 |
| MD RH SLFT | rs35478105 | Chr16 : 77291428 | 7.34E-07 | 0.05 | 0.264 |
| AD RH SLFT | rs1948719 | Chr16 : 77364184 | 1.77E-06 | 0.03 | 0.265 |
| RD LH ATR | rs35478105 | Chr16 : 77291428 | 6.51E-07 | 0.12 | 0.267 |
| AD LH SLFP | rs2650907 | Chr16 : 77359310 | 8.04E-07 | 0.05 | 0.268 |
| MD LH SLFP | rs147053635 | Chr16 : 77284049 | -1.61E-06 | 0.08 | 0.268 |
| RD LH CAB | rs1948719 | Chr16 : 77364184 | 2.65E-06 | 0.03 | 0.269 |
| AD FMinor | rs12935229 | Chr16 : 77294998 | 1.06E-06 | 0.01 | 0.269 |
| AD FMinor | rs12935394 | Chr16 : 77295093 | 1.06E-06 | 0.01 | 0.269 |
| AD FMinor | rs35296483 | Chr16 : 77293121 | 1.06E-06 | 0.01 | 0.269 |
| MD RH CCG | rs757155340 | Chr16 : 77284051 | -1.99E-06 | 0.04 | 0.269 |
| MD RH CCG | rs3743750 | Chr16 : 77289313 | -1.99E-06 | 0.04 | 0.269 |
| MD RH SLFP | rs147053635 | Chr16 : 77284049 | -1.73E-06 | 0.06 | 0.271 |
| MD RH SLFP | rs2650907 | Chr16 : 77359310 | 6.57E-07 | 0.06 | 0.272 |
| FA RH UNC | rs2650907 | Chr16 : 77359310 | -4.27E-04 | 0.08 | 0.272 |
| AD LH CAB | rs3743750 | Chr16 : 77289313 | -5.06E-06 | 0.01 | 0.273 |
| AD LH CAB | rs757155340 | Chr16 : 77284051 | -5.06E-06 | 0.01 | 0.273 |
| FA RH SLFP | rs2650907 | Chr16 : 77359310 | -3.53E-04 | 0.08 | 0.275 |
| AD LH UNC | rs2650907 | Chr16 : 77359310 | 7.51E-07 | 0.02 | 0.276 |
| RD LH SLFP | rs3743750 | Chr16 : 77289313 | -1.75E-06 | 0.10 | 0.277 |
| RD LH SLFP | rs757155340 | Chr16 : 77284051 | -1.75E-06 | 0.10 | 0.277 |
| RD RH SLFT | rs1948719 | Chr16 : 77364184 | 1.10E-06 | 0.06 | 0.277 |
| RD LH SLFP | rs3743749 | Chr16 : 77289338 | -5.74E-07 | 0.10 | 0.279 |
| AD LH CCG | rs3743750 | Chr16 : 77289313 | -3.21E-06 | 0.04 | 0.280 |
| AD LH CCG | rs757155340 | Chr16 : 77284051 | -3.21E-06 | 0.04 | 0.280 |
| FA LH ILF | rs1948719 | Chr16 : 77364184 | -7.50E-04 | 0.06 | 0.282 |
| AD LH SLFP | rs3743749 | Chr16 : 77289338 | -7.75E-07 | 0.06 | 0.287 |
| RD RH SLFP | rs2650907 | Chr16 : 77359310 | 6.12E-07 | 0.08 | 0.288 |
| RD RH UNC | rs3743749 | Chr16 : 77289338 | -6.70E-07 | 0.07 | 0.288 |
| MD LH CST | rs1948719 | Chr16 : 77364184 | 1.26E-06 | 0.07 | 0.290 |
| AD LH CST | rs2650907 | Chr16 : 77359310 | 7.69E-07 | 0.03 | 0.295 |
| FA LH UNC | rs11643211 | Chr16 : 77367648 | -4.87E-04 | 0.09 | 0.296 |
| AD RH SLFP | rs1948719 | Chr16 : 77364184 | 1.53E-06 | 0.04 | 0.297 |
| RD RH SLFT | rs2650907 | Chr16 : 77359310 | 5.35E-07 | 0.06 | 0.298 |
| AD LH SLFP | rs147053635 | Chr16 : 77284049 | -1.98E-06 | 0.04 | 0.299 |
| RD LH SLFP | rs147053635 | Chr16 : 77284049 | -1.42E-06 | 0.10 | 0.303 |
| AD FMajor | rs11643211 | Chr16 : 77367648 | -1.23E-06 | 0.02 | 0.306 |
| AD LH ATR | rs35478105 | Chr16 : 77291428 | 8.29E-07 | 0.04 | 0.306 |
| FA RH CAB | rs757155340 | Chr16 : 77284051 | 1.96E-03 | 0.02 | 0.308 |
| FA RH CAB | rs3743750 | Chr16 : 77289313 | 1.96E-03 | 0.02 | 0.308 |
| FA RH CCG | rs757155340 | Chr16 : 77284051 | 2.25E-03 | 0.03 | 0.308 |
| FA RH CCG | rs3743750 | Chr16 : 77289313 | 2.25E-03 | 0.03 | 0.308 |
| FA FMajor | rs3743749 | Chr16 : 77289338 | 4.92E-04 | 0.04 | 0.309 |
| AD RH SLFP | rs2650907 | Chr16 : 77359310 | 7.48E-07 | 0.04 | 0.313 |
| MD LH CCG | rs12935229 | Chr16 : 77294998 | 6.90E-07 | 0.05 | 0.314 |
| MD LH CCG | rs35296483 | Chr16 : 77293121 | 6.90E-07 | 0.05 | 0.314 |
| MD LH CCG | rs12935394 | Chr16 : 77295093 | 6.90E-07 | 0.05 | 0.314 |
| MD RH SLFP | rs35478105 | Chr16 : 77291428 | 6.86E-07 | 0.06 | 0.315 |
| RD LH ILF | rs2650907 | Chr16 : 77359310 | 5.50E-07 | 0.05 | 0.320 |
| MD RH ATR | rs147053635 | Chr16 : 77284049 | -1.38E-06 | 0.06 | 0.321 |
| FA RH ATR | rs3743750 | Chr16 : 77289313 | 1.04E-03 | 0.08 | 0.324 |
| FA RH ATR | rs757155340 | Chr16 : 77284051 | 1.04E-03 | 0.08 | 0.324 |
| FA RH CCG | rs3743749 | Chr16 : 77289338 | 7.10E-04 | 0.04 | 0.326 |
| FA RH SLFP | rs3743750 | Chr16 : 77289313 | 9.72E-04 | 0.08 | 0.328 |
| FA RH SLFP | rs757155340 | Chr16 : 77284051 | 9.72E-04 | 0.08 | 0.328 |
| FA FMinor | rs1948719 | Chr16 : 77364184 | 8.48E-04 | 0.08 | 0.329 |
| MD FMajor | rs147053635 | Chr16 : 77284049 | -2.02E-06 | 0.02 | 0.329 |
| AD LH ATR | rs2650907 | Chr16 : 77359310 | 6.86E-07 | 0.04 | 0.334 |
| MD RH UNC | rs2650907 | Chr16 : 77359310 | 6.26E-07 | 0.05 | 0.334 |
| FA RH CST | rs3743749 | Chr16 : 77289338 | 3.79E-04 | 0.09 | 0.334 |
| MD LH CAB | rs757155340 | Chr16 : 77284051 | -3.71E-06 | 0.02 | 0.335 |
| MD LH CAB | rs3743750 | Chr16 : 77289313 | -3.71E-06 | 0.02 | 0.335 |
| MD LH ILF | rs2650907 | Chr16 : 77359310 | 5.56E-07 | 0.04 | 0.335 |
| AD RH ATR | rs3743750 | Chr16 : 77289313 | -2.17E-06 | 0.02 | 0.337 |
| AD RH ATR | rs757155340 | Chr16 : 77284051 | -2.17E-06 | 0.02 | 0.337 |
| RD RH CST | rs1948719 | Chr16 : 77364184 | 1.29E-06 | 0.07 | 0.337 |
| AD LH SLFP | rs35478105 | Chr16 : 77291428 | 7.97E-07 | 0.04 | 0.337 |
| MD RH CCG | rs35478105 | Chr16 : 77291428 | 6.40E-07 | 0.04 | 0.341 |
| AD RH SLFT | rs11643211 | Chr16 : 77367648 | -9.54E-07 | 0.03 | 0.341 |
| AD FMajor | rs2650907 | Chr16 : 77359310 | 9.15E-07 | 0.02 | 0.342 |
| MD LH SLFT | rs11643211 | Chr16 : 77367648 | -6.39E-07 | 0.06 | 0.342 |
| AD RH SLFT | rs147053635 | Chr16 : 77284049 | -2.00E-06 | 0.04 | 0.344 |
| RD RH CST | rs3743749 | Chr16 : 77289338 | -6.45E-07 | 0.07 | 0.346 |
| RD RH CAB | rs147053635 | Chr16 : 77284049 | -3.67E-06 | 0.01 | 0.347 |
| FA FMajor | rs147053635 | Chr16 : 77284049 | 1.18E-03 | 0.04 | 0.347 |
| MD RH ILF | rs35478105 | Chr16 : 77291428 | 6.53E-07 | 0.05 | 0.348 |
| RD RH UNC | rs2650907 | Chr16 : 77359310 | 5.85E-07 | 0.07 | 0.349 |
| FA RH CST | rs147053635 | Chr16 : 77284049 | 9.57E-04 | 0.09 | 0.349 |
| MD LH ATR | rs2650907 | Chr16 : 77359310 | 4.93E-07 | 0.10 | 0.352 |
| AD LH CST | rs35296483 | Chr16 : 77293121 | 7.65E-07 | 0.03 | 0.353 |
| AD LH CST | rs12935229 | Chr16 : 77294998 | 7.65E-07 | 0.03 | 0.353 |
| AD LH CST | rs12935394 | Chr16 : 77295093 | 7.65E-07 | 0.03 | 0.353 |
| MD LH ILF | rs147053635 | Chr16 : 77284049 | -1.41E-06 | 0.04 | 0.353 |
| AD RH SLFP | rs12935394 | Chr16 : 77295093 | 7.72E-07 | 0.03 | 0.354 |
| AD RH SLFP | rs12935229 | Chr16 : 77294998 | 7.72E-07 | 0.03 | 0.354 |
| AD RH SLFP | rs35296483 | Chr16 : 77293121 | 7.72E-07 | 0.03 | 0.354 |
| FA LH SLFP | rs1948719 | Chr16 : 77364184 | -5.59E-04 | 0.07 | 0.354 |
| RD FMajor | rs147053635 | Chr16 : 77284049 | -2.06E-06 | 0.03 | 0.358 |
| AD FMajor | rs3743750 | Chr16 : 77289313 | -2.69E-06 | 0.02 | 0.362 |
| AD FMajor | rs757155340 | Chr16 : 77284051 | -2.69E-06 | 0.02 | 0.362 |
| FA RH CAB | rs3743749 | Chr16 : 77289338 | 5.72E-04 | 0.02 | 0.364 |
| AD RH UNC | rs11643211 | Chr16 : 77367648 | -9.72E-07 | 0.02 | 0.367 |
| RD FMajor | rs3743749 | Chr16 : 77289338 | -7.73E-07 | 0.03 | 0.368 |
| MD FMinor | rs2650907 | Chr16 : 77359310 | 4.72E-07 | 0.04 | 0.373 |
| RD FMinor | rs757155340 | Chr16 : 77284051 | -1.47E-06 | 0.07 | 0.374 |
| RD FMinor | rs3743750 | Chr16 : 77289313 | -1.47E-06 | 0.07 | 0.374 |
| RD LH CAB | rs3743749 | Chr16 : 77289338 | -1.08E-06 | 0.03 | 0.377 |
| AD LH ATR | rs757155340 | Chr16 : 77284051 | -1.92E-06 | 0.04 | 0.379 |
| AD LH ATR | rs3743750 | Chr16 : 77289313 | -1.92E-06 | 0.04 | 0.379 |
| FA FMajor | rs11643211 | Chr16 : 77367648 | -5.26E-04 | 0.04 | 0.379 |
| MD LH ATR | rs3743749 | Chr16 : 77289338 | -4.67E-07 | 0.10 | 0.381 |
| AD LH SLFT | rs2650907 | Chr16 : 77359310 | 6.12E-07 | 0.04 | 0.382 |
| RD FMinor | rs3743749 | Chr16 : 77289338 | -4.72E-07 | 0.07 | 0.386 |
| FA LH UNC | rs147053635 | Chr16 : 77284049 | 8.53E-04 | 0.09 | 0.386 |
| RD LH SLFT | rs3743749 | Chr16 : 77289338 | -4.53E-07 | 0.07 | 0.388 |
| RD RH CAB | rs1948719 | Chr16 : 77364184 | 2.52E-06 | 0.01 | 0.391 |
| MD FMinor | rs12935394 | Chr16 : 77295093 | 5.09E-07 | 0.04 | 0.392 |
| MD FMinor | rs35296483 | Chr16 : 77293121 | 5.09E-07 | 0.04 | 0.392 |
| MD FMinor | rs12935229 | Chr16 : 77294998 | 5.09E-07 | 0.04 | 0.392 |
| MD LH CST | rs2650907 | Chr16 : 77359310 | 5.12E-07 | 0.06 | 0.393 |
| MD LH UNC | rs35478105 | Chr16 : 77291428 | 5.00E-07 | 0.07 | 0.395 |
| RD LH UNC | rs147053635 | Chr16 : 77284049 | -1.18E-06 | 0.09 | 0.397 |
| MD LH SLFP | rs2650907 | Chr16 : 77359310 | 4.65E-07 | 0.08 | 0.399 |
| AD RH UNC | rs757155340 | Chr16 : 77284051 | -2.23E-06 | 0.02 | 0.400 |
| AD RH UNC | rs3743750 | Chr16 : 77289313 | -2.23E-06 | 0.02 | 0.400 |
| RD RH UNC | rs35478105 | Chr16 : 77291428 | 6.00E-07 | 0.07 | 0.401 |
| FA LH CCG | rs3743749 | Chr16 : 77289338 | 4.70E-04 | 0.04 | 0.403 |
| MD RH ILF | rs2650907 | Chr16 : 77359310 | 5.10E-07 | 0.05 | 0.403 |
| MD RH CCG | rs2650907 | Chr16 : 77359310 | 4.89E-07 | 0.04 | 0.405 |
| MD RH CST | rs3743749 | Chr16 : 77289338 | -5.84E-07 | 0.06 | 0.406 |
| FA RH SLFT | rs3743750 | Chr16 : 77289313 | 7.28E-04 | 0.05 | 0.407 |
| FA RH SLFT | rs757155340 | Chr16 : 77284051 | 7.28E-04 | 0.05 | 0.407 |
| FA RH UNC | rs1948719 | Chr16 : 77364184 | -6.38E-04 | 0.08 | 0.407 |
| FA FMinor | rs11643211 | Chr16 : 77367648 | -4.53E-04 | 0.07 | 0.408 |
| AD LH UNC | rs12935394 | Chr16 : 77295093 | 6.35E-07 | 0.01 | 0.411 |
| AD LH UNC | rs35296483 | Chr16 : 77293121 | 6.35E-07 | 0.01 | 0.411 |
| AD LH UNC | rs12935229 | Chr16 : 77294998 | 6.35E-07 | 0.01 | 0.411 |
| AD RH UNC | rs2650907 | Chr16 : 77359310 | 7.08E-07 | 0.02 | 0.413 |
| AD LH SLFT | rs147053635 | Chr16 : 77284049 | -1.50E-06 | 0.04 | 0.414 |
| RD LH UNC | rs35478105 | Chr16 : 77291428 | 4.93E-07 | 0.09 | 0.414 |
| FA LH ILF | rs147053635 | Chr16 : 77284049 | 7.56E-04 | 0.05 | 0.416 |
| RD LH CAB | rs757155340 | Chr16 : 77284051 | -3.03E-06 | 0.02 | 0.416 |
| RD LH CAB | rs3743750 | Chr16 : 77289313 | -3.03E-06 | 0.02 | 0.416 |
| AD RH CCG | rs3743749 | Chr16 : 77289338 | 6.21E-07 | 0.01 | 0.417 |
| MD RH SLFT | rs2650907 | Chr16 : 77359310 | 4.65E-07 | 0.05 | 0.420 |
| MD LH SLFT | rs3743749 | Chr16 : 77289338 | -4.37E-07 | 0.07 | 0.421 |
| MD RH SLFP | rs11643211 | Chr16 : 77367648 | -5.99E-07 | 0.06 | 0.423 |
| MD LH CCG | rs757155340 | Chr16 : 77284051 | -1.50E-06 | 0.04 | 0.424 |
| MD LH CCG | rs3743750 | Chr16 : 77289313 | -1.50E-06 | 0.04 | 0.424 |
| FA LH CAB | rs11643211 | Chr16 : 77367648 | -5.58E-04 | 0.04 | 0.428 |
| MD LH UNC | rs11643211 | Chr16 : 77367648 | -5.08E-07 | 0.07 | 0.429 |
| RD RH CCG | rs3743749 | Chr16 : 77289338 | -6.52E-07 | 0.04 | 0.431 |
| FA FMajor | rs1948719 | Chr16 : 77364184 | 7.45E-04 | 0.04 | 0.433 |
| MD FMinor | rs147053635 | Chr16 : 77284049 | -1.09E-06 | 0.04 | 0.433 |
| FA FMinor | rs35478105 | Chr16 : 77291428 | -3.91E-04 | 0.08 | 0.435 |
| MD LH CST | rs3743749 | Chr16 : 77289338 | -4.71E-07 | 0.07 | 0.435 |
| MD RH SLFT | rs11643211 | Chr16 : 77367648 | -5.61E-07 | 0.05 | 0.435 |
| AD RH ILF | rs35296483 | Chr16 : 77293121 | 7.58E-07 | 0.03 | 0.436 |
| AD RH ILF | rs12935394 | Chr16 : 77295093 | 7.58E-07 | 0.03 | 0.436 |
| AD RH ILF | rs12935229 | Chr16 : 77294998 | 7.58E-07 | 0.03 | 0.436 |
| MD LH SLFT | rs2650907 | Chr16 : 77359310 | 4.18E-07 | 0.07 | 0.437 |
| RD LH ATR | rs2650907 | Chr16 : 77359310 | 3.96E-07 | 0.12 | 0.440 |
| MD RH CAB | rs147053635 | Chr16 : 77284049 | -3.08E-06 | 0.01 | 0.441 |
| RD FMinor | rs1948719 | Chr16 : 77364184 | 8.21E-07 | 0.07 | 0.442 |
| AD RH SLFP | rs3743749 | Chr16 : 77289338 | -5.75E-07 | 0.04 | 0.443 |
| AD FMajor | rs147053635 | Chr16 : 77284049 | -1.94E-06 | 0.02 | 0.443 |
| MD LH UNC | rs147053635 | Chr16 : 77284049 | -1.03E-06 | 0.07 | 0.448 |
| FA RH UNC | rs35478105 | Chr16 : 77291428 | -3.33E-04 | 0.08 | 0.454 |
| MD FMajor | rs3743750 | Chr16 : 77289313 | -1.81E-06 | 0.02 | 0.454 |
| MD FMajor | rs757155340 | Chr16 : 77284051 | -1.81E-06 | 0.02 | 0.454 |
| FA RH CST | rs11643211 | Chr16 : 77367648 | -3.61E-04 | 0.09 | 0.457 |
| RD LH CCG | rs3743749 | Chr16 : 77289338 | -4.93E-07 | 0.05 | 0.462 |
| RD FMajor | rs2650907 | Chr16 : 77359310 | -6.25E-07 | 0.03 | 0.463 |
| AD RH UNC | rs147053635 | Chr16 : 77284049 | -1.66E-06 | 0.02 | 0.463 |
| FA RH ILF | rs3743750 | Chr16 : 77289313 | 8.50E-04 | 0.05 | 0.464 |
| FA RH ILF | rs757155340 | Chr16 : 77284051 | 8.50E-04 | 0.05 | 0.464 |
| FA RH CCG | rs35478105 | Chr16 : 77291428 | 5.98E-04 | 0.03 | 0.466 |
| FA LH ILF | rs2650907 | Chr16 : 77359310 | -2.56E-04 | 0.05 | 0.468 |
| FA LH CAB | rs35478105 | Chr16 : 77291428 | -4.65E-04 | 0.04 | 0.471 |
| RD RH ATR | rs147053635 | Chr16 : 77284049 | -9.53E-07 | 0.09 | 0.472 |
| MD RH UNC | rs35478105 | Chr16 : 77291428 | 5.29E-07 | 0.05 | 0.474 |
| RD FMinor | rs147053635 | Chr16 : 77284049 | -1.01E-06 | 0.07 | 0.475 |
| AD LH CCG | rs12935229 | Chr16 : 77294998 | -7.72E-07 | 0.03 | 0.478 |
| AD LH CCG | rs12935394 | Chr16 : 77295093 | -7.72E-07 | 0.03 | 0.478 |
| AD LH CCG | rs35296483 | Chr16 : 77293121 | -7.72E-07 | 0.03 | 0.478 |
| RD RH SLFT | rs3743749 | Chr16 : 77289338 | -3.67E-07 | 0.06 | 0.479 |
| FA LH CST | rs1948719 | Chr16 : 77364184 | 5.34E-04 | 0.09 | 0.482 |
| AD LH ILF | rs2650907 | Chr16 : 77359310 | 5.66E-07 | 0.03 | 0.483 |
| AD RH CCG | rs2650907 | Chr16 : 77359310 | 5.21E-07 | 0.01 | 0.492 |
| RD LH CST | rs147053635 | Chr16 : 77284049 | -1.09E-06 | 0.08 | 0.500 |
| FA FMinor | rs3743749 | Chr16 : 77289338 | 2.99E-04 | 0.07 | 0.501 |
| RD RH CCG | rs147053635 | Chr16 : 77284049 | -1.45E-06 | 0.04 | 0.504 |
| FA RH SLFT | rs11643211 | Chr16 : 77367648 | 2.35E-04 | 0.05 | 0.510 |
| FA LH SLFP | rs147053635 | Chr16 : 77284049 | 5.23E-04 | 0.07 | 0.514 |
| AD RH CAB | rs35478105 | Chr16 : 77291428 | 1.32E-06 | 0.02 | 0.515 |
| FA FMajor | rs35478105 | Chr16 : 77291428 | 3.55E-04 | 0.04 | 0.517 |
| MD LH CAB | rs3743749 | Chr16 : 77289338 | -8.17E-07 | 0.02 | 0.518 |
| RD LH ILF | rs3743749 | Chr16 : 77289338 | -3.55E-07 | 0.04 | 0.526 |
| MD LH UNC | rs2650907 | Chr16 : 77359310 | 3.26E-07 | 0.06 | 0.527 |
| AD RH CST | rs12935394 | Chr16 : 77295093 | 6.09E-07 | 0.03 | 0.528 |
| AD RH CST | rs12935229 | Chr16 : 77294998 | 6.09E-07 | 0.03 | 0.528 |
| AD RH CST | rs35296483 | Chr16 : 77293121 | 6.09E-07 | 0.03 | 0.528 |
| FA RH ATR | rs1948719 | Chr16 : 77364184 | -4.25E-04 | 0.09 | 0.531 |
| MD RH CCG | rs147053635 | Chr16 : 77284049 | -9.66E-07 | 0.04 | 0.532 |
| AD LH CST | rs35478105 | Chr16 : 77291428 | 5.25E-07 | 0.03 | 0.532 |
| RD LH CST | rs2650907 | Chr16 : 77359310 | 3.84E-07 | 0.08 | 0.534 |
| RD LH SLFT | rs2650907 | Chr16 : 77359310 | 3.22E-07 | 0.07 | 0.535 |
| AD LH CCG | rs147053635 | Chr16 : 77284049 | 1.58E-06 | 0.03 | 0.536 |
| FA RH ILF | rs1948719 | Chr16 : 77364184 | -4.59E-04 | 0.06 | 0.538 |
| AD LH UNC | rs35478105 | Chr16 : 77291428 | 4.83E-07 | 0.01 | 0.540 |
| AD FMinor | rs11643211 | Chr16 : 77367648 | -6.54E-07 | 0.00 | 0.542 |
| FA RH CCG | rs1948719 | Chr16 : 77364184 | -8.62E-04 | 0.03 | 0.543 |
| FA LH UNC | rs3743750 | Chr16 : 77289313 | 6.99E-04 | 0.09 | 0.543 |
| FA LH UNC | rs757155340 | Chr16 : 77284051 | 6.99E-04 | 0.09 | 0.543 |
| FA RH CAB | rs11643211 | Chr16 : 77367648 | -4.72E-04 | 0.02 | 0.545 |
| MD FMajor | rs3743749 | Chr16 : 77289338 | -4.82E-07 | 0.02 | 0.545 |
| MD FMinor | rs3743749 | Chr16 : 77289338 | -3.21E-07 | 0.04 | 0.549 |
| FA RH SLFT | rs3743749 | Chr16 : 77289338 | 1.72E-04 | 0.05 | 0.550 |
| AD RH CAB | rs2650907 | Chr16 : 77359310 | 1.06E-06 | 0.02 | 0.551 |
| AD RH CCG | rs11643211 | Chr16 : 77367648 | -5.63E-07 | 0.01 | 0.551 |
| AD LH CAB | rs11643211 | Chr16 : 77367648 | -1.12E-06 | 0.01 | 0.552 |
| FA RH ATR | rs2650907 | Chr16 : 77359310 | -2.03E-04 | 0.08 | 0.555 |
| FA LH UNC | rs35478105 | Chr16 : 77291428 | -2.50E-04 | 0.09 | 0.559 |
| FA RH CCG | rs147053635 | Chr16 : 77284049 | 1.10E-03 | 0.03 | 0.560 |
| AD LH SLFT | rs3743749 | Chr16 : 77289338 | -4.06E-07 | 0.05 | 0.564 |
| RD RH CCG | rs2650907 | Chr16 : 77359310 | 4.73E-07 | 0.04 | 0.565 |
| AD RH CST | rs11643211 | Chr16 : 77367648 | -6.16E-07 | 0.03 | 0.567 |
| RD RH SLFT | rs11643211 | Chr16 : 77367648 | -3.64E-07 | 0.06 | 0.571 |
| AD FMinor | rs147053635 | Chr16 : 77284049 | -1.28E-06 | 0.00 | 0.572 |
| RD LH SLFP | rs2650907 | Chr16 : 77359310 | 2.96E-07 | 0.10 | 0.572 |
| FA LH ILF | rs3743749 | Chr16 : 77289338 | 1.99E-04 | 0.05 | 0.577 |
| MD RH UNC | rs3743749 | Chr16 : 77289338 | -3.64E-07 | 0.05 | 0.577 |
| AD RH SLFP | rs147053635 | Chr16 : 77284049 | -1.07E-06 | 0.03 | 0.585 |
| FA LH CCG | rs147053635 | Chr16 : 77284049 | 7.98E-04 | 0.04 | 0.585 |
| FA RH CST | rs2650907 | Chr16 : 77359310 | -2.10E-04 | 0.09 | 0.589 |
| MD LH ILF | rs3743749 | Chr16 : 77289338 | -3.12E-07 | 0.04 | 0.592 |
| AD RH CST | rs3743749 | Chr16 : 77289338 | -4.63E-07 | 0.03 | 0.594 |
| RD RH CAB | rs11643211 | Chr16 : 77367648 | 9.80E-07 | 0.01 | 0.597 |
| FA RH SLFT | rs1948719 | Chr16 : 77364184 | -2.97E-04 | 0.06 | 0.599 |
| RD FMajor | rs3743750 | Chr16 : 77289313 | -1.37E-06 | 0.03 | 0.601 |
| RD FMajor | rs757155340 | Chr16 : 77284051 | -1.37E-06 | 0.03 | 0.601 |
| FA FMajor | rs35296483 | Chr16 : 77293121 | -2.76E-04 | 0.04 | 0.607 |
| FA FMajor | rs12935229 | Chr16 : 77294998 | -2.76E-04 | 0.04 | 0.607 |
| FA FMajor | rs12935394 | Chr16 : 77295093 | -2.76E-04 | 0.04 | 0.607 |
| AD RH ATR | rs35478105 | Chr16 : 77291428 | 4.28E-07 | 0.02 | 0.610 |
| AD LH SLFP | rs35296483 | Chr16 : 77293121 | 4.03E-07 | 0.04 | 0.621 |
| AD LH SLFP | rs12935229 | Chr16 : 77294998 | 4.03E-07 | 0.04 | 0.621 |
| AD LH SLFP | rs12935394 | Chr16 : 77295093 | 4.03E-07 | 0.04 | 0.621 |
| RD RH ATR | rs2650907 | Chr16 : 77359310 | 2.42E-07 | 0.09 | 0.631 |
| MD RH SLFT | rs3743749 | Chr16 : 77289338 | -2.76E-07 | 0.05 | 0.634 |
| FA LH ATR | rs35478105 | Chr16 : 77291428 | -1.75E-04 | 0.11 | 0.640 |
| FA RH ATR | rs11643211 | Chr16 : 77367648 | 1.99E-04 | 0.08 | 0.643 |
| AD RH SLFT | rs12935394 | Chr16 : 77295093 | 4.16E-07 | 0.03 | 0.645 |
| AD RH SLFT | rs12935229 | Chr16 : 77294998 | 4.16E-07 | 0.03 | 0.645 |
| AD RH SLFT | rs35296483 | Chr16 : 77293121 | 4.16E-07 | 0.03 | 0.645 |
| MD FMajor | rs35478105 | Chr16 : 77291428 | 4.13E-07 | 0.02 | 0.646 |
| RD RH CST | rs147053635 | Chr16 : 77284049 | -8.20E-07 | 0.07 | 0.646 |
| RD FMajor | rs1948719 | Chr16 : 77364184 | 7.70E-07 | 0.03 | 0.647 |
| FA LH CAB | rs1948719 | Chr16 : 77364184 | -5.09E-04 | 0.05 | 0.648 |
| RD LH SLFT | rs11643211 | Chr16 : 77367648 | -2.94E-07 | 0.07 | 0.650 |
| AD LH ATR | rs3743749 | Chr16 : 77289338 | 3.22E-07 | 0.04 | 0.652 |
| AD LH ILF | rs147053635 | Chr16 : 77284049 | -9.43E-07 | 0.03 | 0.657 |
| FA RH CAB | rs12935394 | Chr16 : 77295093 | 3.10E-04 | 0.02 | 0.658 |
| FA RH CAB | rs12935229 | Chr16 : 77294998 | 3.10E-04 | 0.02 | 0.658 |
| FA RH CAB | rs35296483 | Chr16 : 77293121 | 3.10E-04 | 0.02 | 0.658 |
| AD LH SLFT | rs12935229 | Chr16 : 77294998 | 3.46E-07 | 0.04 | 0.660 |
| AD LH SLFT | rs35296483 | Chr16 : 77293121 | 3.46E-07 | 0.04 | 0.660 |
| AD LH SLFT | rs12935394 | Chr16 : 77295093 | 3.46E-07 | 0.04 | 0.660 |
| RD LH CAB | rs147053635 | Chr16 : 77284049 | -1.40E-06 | 0.02 | 0.661 |
| AD LH CAB | rs2650907 | Chr16 : 77359310 | 6.53E-07 | 0.01 | 0.663 |
| FA LH ILF | rs757155340 | Chr16 : 77284051 | 4.69E-04 | 0.06 | 0.666 |
| FA LH ILF | rs3743750 | Chr16 : 77289313 | 4.69E-04 | 0.06 | 0.666 |
| MD RH UNC | rs11643211 | Chr16 : 77367648 | -3.47E-07 | 0.05 | 0.668 |
| MD RH CAB | rs2650907 | Chr16 : 77359310 | 6.50E-07 | 0.01 | 0.669 |
| AD LH CST | rs3743749 | Chr16 : 77289338 | 3.17E-07 | 0.03 | 0.669 |
| MD RH ATR | rs2650907 | Chr16 : 77359310 | 2.24E-07 | 0.06 | 0.671 |
| AD LH ATR | rs11643211 | Chr16 : 77367648 | -3.75E-07 | 0.04 | 0.672 |
| RD FMinor | rs11643211 | Chr16 : 77367648 | 2.84E-07 | 0.07 | 0.673 |
| FA LH SLFT | rs3743749 | Chr16 : 77289338 | 1.27E-04 | 0.04 | 0.675 |
| FA LH SLFT | rs11643211 | Chr16 : 77367648 | -1.55E-04 | 0.04 | 0.678 |
| AD RH SLFT | rs35478105 | Chr16 : 77291428 | 3.80E-07 | 0.03 | 0.679 |
| RD LH CST | rs1948719 | Chr16 : 77364184 | 5.05E-07 | 0.08 | 0.680 |
| AD FMajor | rs12935229 | Chr16 : 77294998 | 4.45E-07 | 0.02 | 0.680 |
| AD FMajor | rs12935394 | Chr16 : 77295093 | 4.45E-07 | 0.02 | 0.680 |
| AD FMajor | rs35296483 | Chr16 : 77293121 | 4.45E-07 | 0.02 | 0.680 |
| RD RH ATR | rs11643211 | Chr16 : 77367648 | -2.57E-07 | 0.08 | 0.683 |
| FA LH SLFT | rs3743750 | Chr16 : 77289313 | 3.72E-04 | 0.04 | 0.686 |
| FA LH SLFT | rs757155340 | Chr16 : 77284051 | 3.72E-04 | 0.04 | 0.686 |
| FA FMinor | rs147053635 | Chr16 : 77284049 | 4.66E-04 | 0.07 | 0.687 |
| AD RH CAB | rs147053635 | Chr16 : 77284049 | -1.89E-06 | 0.02 | 0.687 |
| AD RH SLFT | rs2650907 | Chr16 : 77359310 | 3.23E-07 | 0.03 | 0.688 |
| FA FMinor | rs12935229 | Chr16 : 77294998 | 1.94E-04 | 0.07 | 0.694 |
| FA FMinor | rs35296483 | Chr16 : 77293121 | 1.94E-04 | 0.07 | 0.694 |
| FA FMinor | rs12935394 | Chr16 : 77295093 | 1.94E-04 | 0.07 | 0.694 |
| AD RH UNC | rs35478105 | Chr16 : 77291428 | 3.88E-07 | 0.02 | 0.694 |
| RD LH CAB | rs35478105 | Chr16 : 77291428 | 5.39E-07 | 0.02 | 0.697 |
| MD RH CST | rs35478105 | Chr16 : 77291428 | 3.09E-07 | 0.05 | 0.698 |
| FA LH CCG | rs757155340 | Chr16 : 77284051 | -6.58E-04 | 0.05 | 0.699 |
| FA LH CCG | rs3743750 | Chr16 : 77289313 | -6.58E-04 | 0.05 | 0.699 |
| MD RH CCG | rs11643211 | Chr16 : 77367648 | -2.83E-07 | 0.04 | 0.700 |
| FA LH CAB | rs35296483 | Chr16 : 77293121 | 2.44E-04 | 0.04 | 0.701 |
| FA LH CAB | rs12935394 | Chr16 : 77295093 | 2.44E-04 | 0.04 | 0.701 |
| FA LH CAB | rs12935229 | Chr16 : 77294998 | 2.44E-04 | 0.04 | 0.701 |
| MD RH CCG | rs3743749 | Chr16 : 77289338 | -2.28E-07 | 0.04 | 0.701 |
| RD FMinor | rs12935229 | Chr16 : 77294998 | 2.32E-07 | 0.07 | 0.702 |
| RD FMinor | rs12935394 | Chr16 : 77295093 | 2.32E-07 | 0.07 | 0.702 |
| RD FMinor | rs35296483 | Chr16 : 77293121 | 2.32E-07 | 0.07 | 0.702 |
| FA LH SLFT | rs2650907 | Chr16 : 77359310 | -1.14E-04 | 0.04 | 0.703 |
| RD RH CST | rs2650907 | Chr16 : 77359310 | 2.57E-07 | 0.07 | 0.705 |
| MD FMajor | rs12935394 | Chr16 : 77295093 | 3.33E-07 | 0.02 | 0.705 |
| MD FMajor | rs12935229 | Chr16 : 77294998 | 3.33E-07 | 0.02 | 0.705 |
| MD FMajor | rs35296483 | Chr16 : 77293121 | 3.33E-07 | 0.02 | 0.705 |
| AD LH UNC | rs3743749 | Chr16 : 77289338 | -2.63E-07 | 0.01 | 0.706 |
| AD LH CST | rs11643211 | Chr16 : 77367648 | -3.45E-07 | 0.03 | 0.707 |
| MD RH ATR | rs11643211 | Chr16 : 77367648 | -2.47E-07 | 0.06 | 0.707 |
| RD RH CST | rs35478105 | Chr16 : 77291428 | 2.90E-07 | 0.07 | 0.708 |
| RD FMajor | rs11643211 | Chr16 : 77367648 | 3.96E-07 | 0.03 | 0.709 |
| RD RH CST | rs11643211 | Chr16 : 77367648 | 3.11E-07 | 0.07 | 0.714 |
| MD RH CST | rs2650907 | Chr16 : 77359310 | 2.55E-07 | 0.05 | 0.715 |
| FA LH ATR | rs2650907 | Chr16 : 77359310 | -1.20E-04 | 0.10 | 0.716 |
| MD LH CAB | rs2650907 | Chr16 : 77359310 | 4.53E-07 | 0.02 | 0.717 |
| MD LH CST | rs147053635 | Chr16 : 77284049 | -5.69E-07 | 0.06 | 0.719 |
| MD LH CCG | rs3743749 | Chr16 : 77289338 | -2.19E-07 | 0.05 | 0.722 |
| AD RH CST | rs35478105 | Chr16 : 77291428 | 3.45E-07 | 0.03 | 0.726 |
| RD FMinor | rs2650907 | Chr16 : 77359310 | 1.86E-07 | 0.07 | 0.730 |
| MD LH SLFP | rs11643211 | Chr16 : 77367648 | -2.38E-07 | 0.08 | 0.730 |
| FA LH CST | rs11643211 | Chr16 : 77367648 | -1.64E-04 | 0.09 | 0.732 |
| AD LH CCG | rs3743749 | Chr16 : 77289338 | 3.28E-07 | 0.03 | 0.738 |
| FA LH ILF | rs11643211 | Chr16 : 77367648 | 1.46E-04 | 0.06 | 0.739 |
| AD RH ILF | rs35478105 | Chr16 : 77291428 | 3.16E-07 | 0.02 | 0.750 |
| RD LH CCG | rs757155340 | Chr16 : 77284051 | -6.43E-07 | 0.04 | 0.753 |
| RD LH CCG | rs3743750 | Chr16 : 77289313 | -6.43E-07 | 0.04 | 0.753 |
| AD LH ATR | rs12935229 | Chr16 : 77294998 | 2.50E-07 | 0.04 | 0.754 |
| AD LH ATR | rs12935394 | Chr16 : 77295093 | 2.50E-07 | 0.04 | 0.754 |
| AD LH ATR | rs35296483 | Chr16 : 77293121 | 2.50E-07 | 0.04 | 0.754 |
| MD RH CAB | rs11643211 | Chr16 : 77367648 | 5.87E-07 | 0.01 | 0.757 |
| RD RH CAB | rs2650907 | Chr16 : 77359310 | 4.44E-07 | 0.01 | 0.765 |
| FA LH CCG | rs35478105 | Chr16 : 77291428 | 1.89E-04 | 0.04 | 0.765 |
| RD RH SLFP | rs11643211 | Chr16 : 77367648 | -2.12E-07 | 0.07 | 0.769 |
| AD RH ILF | rs147053635 | Chr16 : 77284049 | -6.70E-07 | 0.02 | 0.769 |
| RD LH CAB | rs2650907 | Chr16 : 77359310 | 3.53E-07 | 0.03 | 0.771 |
| RD FMajor | rs12935229 | Chr16 : 77294998 | 2.77E-07 | 0.03 | 0.771 |
| RD FMajor | rs12935394 | Chr16 : 77295093 | 2.77E-07 | 0.03 | 0.771 |
| RD FMajor | rs35296483 | Chr16 : 77293121 | 2.77E-07 | 0.03 | 0.771 |
| AD RH CST | rs2650907 | Chr16 : 77359310 | 2.49E-07 | 0.03 | 0.772 |
| MD LH ATR | rs11643211 | Chr16 : 77367648 | -1.90E-07 | 0.09 | 0.775 |
| AD RH UNC | rs3743749 | Chr16 : 77289338 | 2.48E-07 | 0.02 | 0.776 |
| FA LH UNC | rs2650907 | Chr16 : 77359310 | 1.04E-04 | 0.09 | 0.781 |
| AD LH UNC | rs147053635 | Chr16 : 77284049 | -5.02E-07 | 0.01 | 0.782 |
| FA LH SLFT | rs1948719 | Chr16 : 77364184 | 1.64E-04 | 0.04 | 0.782 |
| AD LH ILF | rs3743749 | Chr16 : 77289338 | -2.24E-07 | 0.03 | 0.783 |
| FA LH SLFP | rs3743749 | Chr16 : 77289338 | 8.43E-05 | 0.07 | 0.784 |
| RD LH SLFP | rs11643211 | Chr16 : 77367648 | 1.79E-07 | 0.10 | 0.785 |
| FA RH ILF | rs11643211 | Chr16 : 77367648 | 1.23E-04 | 0.05 | 0.795 |
| FA FMinor | rs2650907 | Chr16 : 77359310 | 1.14E-04 | 0.07 | 0.795 |
| FA FMajor | rs3743750 | Chr16 : 77289313 | 3.78E-04 | 0.04 | 0.797 |
| FA FMajor | rs757155340 | Chr16 : 77284051 | 3.78E-04 | 0.04 | 0.797 |
| AD RH ATR | rs2650907 | Chr16 : 77359310 | 1.88E-07 | 0.02 | 0.798 |
| MD LH CCG | rs11643211 | Chr16 : 77367648 | -1.95E-07 | 0.04 | 0.798 |
| AD LH CST | rs147053635 | Chr16 : 77284049 | 4.83E-07 | 0.03 | 0.803 |
| AD RH ATR | rs11643211 | Chr16 : 77367648 | -2.28E-07 | 0.02 | 0.804 |
| MD RH CST | rs147053635 | Chr16 : 77284049 | -4.53E-07 | 0.05 | 0.805 |
| AD LH CAB | rs147053635 | Chr16 : 77284049 | 9.71E-07 | 0.01 | 0.806 |
| FA LH CCG | rs2650907 | Chr16 : 77359310 | 1.36E-04 | 0.04 | 0.806 |
| FA RH CCG | rs2650907 | Chr16 : 77359310 | -1.74E-04 | 0.03 | 0.808 |
| FA LH CST | rs2650907 | Chr16 : 77359310 | -9.27E-05 | 0.09 | 0.809 |
| FA RH UNC | rs11643211 | Chr16 : 77367648 | -1.17E-04 | 0.07 | 0.811 |
| FA LH CCG | rs11643211 | Chr16 : 77367648 | 1.66E-04 | 0.04 | 0.811 |
| AD LH ATR | rs147053635 | Chr16 : 77284049 | -4.37E-07 | 0.04 | 0.815 |
| AD RH ILF | rs3743749 | Chr16 : 77289338 | 2.04E-07 | 0.03 | 0.816 |
| MD RH ILF | rs3743749 | Chr16 : 77289338 | 1.42E-07 | 0.05 | 0.817 |
| RD LH CCG | rs147053635 | Chr16 : 77284049 | -4.03E-07 | 0.04 | 0.818 |
| AD LH CAB | rs35478105 | Chr16 : 77291428 | -3.96E-07 | 0.01 | 0.818 |
| RD LH CST | rs11643211 | Chr16 : 77367648 | 1.76E-07 | 0.08 | 0.819 |
| AD LH CCG | rs2650907 | Chr16 : 77359310 | 2.21E-07 | 0.03 | 0.820 |
| RD LH UNC | rs2650907 | Chr16 : 77359310 | 1.19E-07 | 0.09 | 0.822 |
| FA RH ATR | rs147053635 | Chr16 : 77284049 | -1.99E-04 | 0.08 | 0.826 |
| AD LH CCG | rs11643211 | Chr16 : 77367648 | -2.59E-07 | 0.03 | 0.831 |
| RD LH CCG | rs11643211 | Chr16 : 77367648 | -1.64E-07 | 0.04 | 0.844 |
| FA RH SLFP | rs11643211 | Chr16 : 77367648 | -7.83E-05 | 0.07 | 0.846 |
| AD LH CAB | rs3743749 | Chr16 : 77289338 | -2.88E-07 | 0.01 | 0.849 |
| RD RH ILF | rs3743749 | Chr16 : 77289338 | 1.11E-07 | 0.06 | 0.850 |
| AD RH ILF | rs2650907 | Chr16 : 77359310 | 1.61E-07 | 0.03 | 0.852 |
| MD LH CAB | rs147053635 | Chr16 : 77284049 | -6.08E-07 | 0.02 | 0.853 |
| MD RH ILF | rs11643211 | Chr16 : 77367648 | -1.38E-07 | 0.04 | 0.856 |
| AD RH ILF | rs11643211 | Chr16 : 77367648 | -1.94E-07 | 0.03 | 0.858 |
| FA LH UNC | rs1948719 | Chr16 : 77364184 | -1.32E-04 | 0.09 | 0.859 |
| AD RH CCG | rs757155340 | Chr16 : 77284051 | 4.04E-07 | 0.01 | 0.862 |
| AD RH CCG | rs3743750 | Chr16 : 77289313 | 4.04E-07 | 0.01 | 0.862 |
| MD LH CCG | rs147053635 | Chr16 : 77284049 | 2.59E-07 | 0.04 | 0.872 |
| MD LH CAB | rs35478105 | Chr16 : 77291428 | 2.27E-07 | 0.02 | 0.873 |
| MD LH CAB | rs11643211 | Chr16 : 77367648 | -2.43E-07 | 0.02 | 0.876 |
| AD RH ATR | rs3743749 | Chr16 : 77289338 | -1.14E-07 | 0.03 | 0.878 |
| RD RH ILF | rs11643211 | Chr16 : 77367648 | -1.10E-07 | 0.05 | 0.880 |
| RD LH ATR | rs11643211 | Chr16 : 77367648 | -9.70E-08 | 0.11 | 0.880 |
| MD FMajor | rs11643211 | Chr16 : 77367648 | -1.46E-07 | 0.02 | 0.882 |
| FA FMinor | rs3743750 | Chr16 : 77289313 | -1.99E-04 | 0.07 | 0.883 |
| FA FMinor | rs757155340 | Chr16 : 77284051 | -1.99E-04 | 0.07 | 0.883 |
| FA LH CAB | rs3743750 | Chr16 : 77289313 | 2.49E-04 | 0.04 | 0.886 |
| FA LH CAB | rs757155340 | Chr16 : 77284051 | 2.49E-04 | 0.04 | 0.886 |
| MD LH ATR | rs147053635 | Chr16 : 77284049 | -1.98E-07 | 0.09 | 0.887 |
| MD FMajor | rs2650907 | Chr16 : 77359310 | -1.12E-07 | 0.02 | 0.887 |
| RD RH CCG | rs11643211 | Chr16 : 77367648 | -1.43E-07 | 0.03 | 0.889 |
| RD LH CAB | rs11643211 | Chr16 : 77367648 | 1.94E-07 | 0.02 | 0.898 |
| FA RH CAB | rs1948719 | Chr16 : 77364184 | -1.56E-04 | 0.02 | 0.899 |
| AD RH CST | rs147053635 | Chr16 : 77284049 | 2.82E-07 | 0.03 | 0.901 |
| FA LH CCG | rs1948719 | Chr16 : 77364184 | -1.36E-04 | 0.04 | 0.901 |
| AD RH SLFP | rs35478105 | Chr16 : 77291428 | -9.88E-08 | 0.03 | 0.907 |
| AD RH SLFT | rs3743749 | Chr16 : 77289338 | -9.38E-08 | 0.04 | 0.908 |
| FA RH CAB | rs2650907 | Chr16 : 77359310 | 6.84E-05 | 0.02 | 0.913 |
| RD LH CCG | rs2650907 | Chr16 : 77359310 | -7.27E-08 | 0.04 | 0.913 |
| AD FMajor | rs3743749 | Chr16 : 77289338 | 1.01E-07 | 0.02 | 0.917 |
| FA LH ATR | rs1948719 | Chr16 : 77364184 | 6.74E-05 | 0.10 | 0.918 |
| FA LH SLFP | rs2650907 | Chr16 : 77359310 | -3.13E-05 | 0.07 | 0.918 |
| RD RH CCG | rs35478105 | Chr16 : 77291428 | 9.38E-08 | 0.03 | 0.921 |
| AD RH CAB | rs11643211 | Chr16 : 77367648 | -1.99E-07 | 0.02 | 0.929 |
| FA LH SLFP | rs3743750 | Chr16 : 77289313 | 7.95E-05 | 0.07 | 0.932 |
| FA LH SLFP | rs757155340 | Chr16 : 77284051 | 7.95E-05 | 0.07 | 0.932 |
| AD RH ATR | rs12935229 | Chr16 : 77294998 | -6.90E-08 | 0.02 | 0.933 |
| AD RH ATR | rs35296483 | Chr16 : 77293121 | -6.90E-08 | 0.02 | 0.933 |
| AD RH ATR | rs12935394 | Chr16 : 77295093 | -6.90E-08 | 0.02 | 0.933 |
| FA RH CCG | rs11643211 | Chr16 : 77367648 | 5.55E-05 | 0.03 | 0.951 |
| AD LH CCG | rs35478105 | Chr16 : 77291428 | 6.66E-08 | 0.03 | 0.952 |
| FA LH ATR | rs147053635 | Chr16 : 77284049 | -5.16E-05 | 0.10 | 0.953 |
| RD LH ATR | rs147053635 | Chr16 : 77284049 | -7.80E-08 | 0.11 | 0.954 |
| MD LH CCG | rs35478105 | Chr16 : 77291428 | 3.92E-08 | 0.04 | 0.955 |
| FA RH CST | rs35478105 | Chr16 : 77291428 | -2.24E-05 | 0.09 | 0.960 |
| RD RH UNC | rs11643211 | Chr16 : 77367648 | -3.48E-08 | 0.07 | 0.964 |
| MD FMinor | rs11643211 | Chr16 : 77367648 | -2.83E-08 | 0.04 | 0.966 |
| MD LH CCG | rs2650907 | Chr16 : 77359310 | 2.52E-08 | 0.05 | 0.967 |
| RD LH CCG | rs35478105 | Chr16 : 77291428 | 2.55E-08 | 0.04 | 0.973 |
| RD FMajor | rs35478105 | Chr16 : 77291428 | -3.10E-08 | 0.03 | 0.975 |
| FA LH CAB | rs2650907 | Chr16 : 77359310 | 1.53E-05 | 0.04 | 0.978 |
| FA LH ATR | rs11643211 | Chr16 : 77367648 | -1.06E-05 | 0.10 | 0.980 |
| FA RH CST | rs1948719 | Chr16 : 77364184 | -1.59E-05 | 0.09 | 0.984 |
| AD FMinor | rs3743749 | Chr16 : 77289338 | -1.68E-08 | 0.00 | 0.985 |
| AD LH ILF | rs35478105 | Chr16 : 77291428 | -6.24E-09 | 0.03 | 0.995 |
| AD RH CCG | rs147053635 | Chr16 : 77284049 | -7.46E-09 | 0.01 | 0.997 |
| RD LH UNC | rs11643211 | Chr16 : 77367648 | -2.20E-09 | 0.09 | 0.997 |
| MD LH CST | rs11643211 | Chr16 : 77367648 | 2.41E-09 | 0.06 | 0.997 |
| FA RH ILF | rs3743749 | Chr16 : 77289338 | -9.94E-07 | 0.05 | 0.998 |
| MD RH CST | rs11643211 | Chr16 : 77367648 | 2.05E-09 | 0.05 | 0.998 |

Linear regression models were fitted for each tract–SNP pair, including genotype, Hamilton Depression Rating Scale (HDRS-17) score, and their interaction term. Age and sex were included as covariates. Reported statistics include unstandardized beta coefficients (β), model R² values, and uncorrected p-values. Nominally significant results (p < 0.05, uncorrected) are highlighted in boldface. No results survived false discovery rate (FDR) correction. Genomic positions are based on the human reference genome build GRCh38.

Abbreviations: AD, axial diffusivity; ATR, anterior thalamic radiation; CAB, cingulum angular bundle; CCG, cingulum cingulate gyrus bundle; CST, corticospinal tract; FA, fractional anisotropy; FMajor, forceps major; FMinor, forceps minor; ILF, inferior longitudinal fasciculus; LH, left hemisphere; MD, mean diffusivity; RD, radial diffusivity; RH, right hemisphere; SLFP, superior longitudinal fasciculus–parietal bundle; SLFT, superior longitudinal fasciculus–temporal bundle; UNC, uncinate fasciculus.

**Supplementary Figure 1. Axial diffusivity in the left cingulum–cingulate gyrus bundle by rs1948719 genotype**

Violin plots show axial diffusivity (AD) values in the left cingulum–cingulate gyrus bundle (CCG), stratified by rs1948719 genotype (TT vs TC/CC) across all participants. Individual data points are overlaid; boxes indicate the median and interquartile range.

**Supplementary Figure 2. Axial diffusivity in the left inferior longitudinal fasciculus by rs11643211 genotype**

Violin plots depict the distribution of axial diffusivity (AD) values in the left inferior longitudinal fasciculus (ILF), stratified by rs11643211 genotype (GG vs AG/AA) and collapsed across diagnostic categories. Individual data points are overlaid; boxes indicate the median and interquartile range.
